# Obesity-related complications, healthcare resource use and weight loss strategies in six European countries: the RESOURCE survey

**DOI:** 10.1038/s41366-023-01325-1

**Published:** 2023-05-31

**Authors:** Marc Evans, Joanna de Courcy, Elisabeth de Laguiche, Mads Faurby, Christiane Lundegaard Haase, Kasper Sommer Matthiessen, Adam Moore, Jonathan Pearson-Stuttard

**Affiliations:** 1 University Hospital Llandough, Penarth, Cardiff, UK; 2 Adelphi Real World, Macclesfield, UK; 3 Novo Nordisk A/S, Søborg, Denmark; 4 Lane Clark & Peacock LLP, London, UK; 5 Department of Epidemiology and Biostatistics, School of Public Health, Imperial College London, London, UK

**Keywords:** Epidemiology, Weight management, Health policy

## Abstract

**Background:**

Obesity-related complications (ORCs), such as type 2 diabetes (T2D) and cardiovascular disease, contribute considerably to the clinical and economic impacts of obesity. To obtain a holistic overview of health and weight management attempts for people with obesity in Europe, we designed the cross-sectional RESOURCE survey to collect data on comorbidities, healthcare resource use (HCRU) and weight loss strategies from people with obesity in France, Germany, Italy, Spain, Sweden and the UK.

**Methods:**

Adults (≥18 years old) with self-reported body mass index (BMI) ≥30 kg/m^2^ who reported interacting with primary or secondary healthcare services in the past 12 months, but had not been pregnant during this time, were recruited from an existing consumer research panel. All data were self-reported via an online survey (May–June 2021). Weight changes over the past year were calculated from participants’ estimated weights.

**Results:**

Of the 1850 participants in the survey, 26.3% reported that they had ≥3 ORCs from a set of 15 conditions of interest. The most frequently reported ORCs were hypertension (39.3% of participants), dyslipidaemia (22.8%) and T2D (17.5%). Participants in obesity class III (BMI 40 to <70 kg/m^2^) were more likely to report multiple ORCs than those in lower obesity classes. The presence of multiple ORCs was linked to various types of HCRU, including a significantly increased chance of reporting hospitalization in the past year. Most participants (78.6%) had attempted to lose weight in the past year, but of those who also reported estimated weight changes, 73.4% had not experienced clinically meaningful weight loss of ≥5%.

**Conclusions:**

ORCs are common in people with obesity, and are linked to increased HCRU. Together with the low reported success rate of weight loss attempts, this highlights an unmet need in Europe for enhanced weight management support for people with obesity.

## Introduction

The prevalence of obesity has increased in the past four decades both worldwide [1] and in Europe [2], and addressing this challenge has become a major public health priority. In 2014, the prevalence of obesity in adults across the EU was estimated as 16% [2], and in 2022 the World Health Organization (WHO) estimated that 23% of adults in the WHO European region were living with obesity [3]. Recent projections suggest that the prevalence of obesity in Europe will continue to increase over the next 10 years [4]. This burgeoning public health issue is reflected in healthcare expenditure: it has been estimated that, on average, EU28 member states will spend more than 8% of national healthcare budgets on overweight, obesity and related conditions between 2020 and 2050 [5].

A systematic literature review of studies published up to 2015 found that approximately 42% of people in the general population worldwide tried to lose weight and 23% tried actively to maintain weight, to avoid weight gain, and people with overweight or obesity were more likely to attempt weight loss than the general population [6]. Whereas many people made dietary and lifestyle adjustments, only 10% of individuals, surveyed across 14 studies, reported using weight loss pills or supplements [6].

As emphasized in the 2022 report by the WHO, obesity has severe clinical and public health implications [3]. High body mass index (BMI) is associated with the risk of developing a broad set of comorbidities, termed obesity-related complications (ORCs), which can affect all organ systems. ORCs include cardiovascular disease (CVD); metabolic complications, such as type 2 diabetes (T2D); respiratory conditions, including asthma and obstructive sleep apnoea; and disorders affecting mobility, such as osteoarthritis and pain [7]. Consequently, obesity is a risk factor for multiple deleterious clinical outcomes, including limitations to activities of daily living [8] and health-related quality of life [9], requirement for long-term treatment of cardiovascular risk factors, and the morbidity and mortality associated with T2D and CVD [10]. ORCs are a major driver of obesity-related costs [11], and increasing BMI class is closely linked to both the risks of experiencing particular ORCs [12] and the costs associated with treating these conditions [7].

Examining the relationships between BMI, ORCs and healthcare resource use (HCRU) is important to identify factors that contribute to costs in obesity. Together with data on attempts to lose weight, this information can be used to identify areas of unmet need in which clinical and economic outcomes could be improved by timely support with weight management. To drive understanding of these challenges, we conducted a cross-sectional survey of people with different classes of obesity, focusing on ORCs, HCRU and weight loss strategies across six European countries.

## Methods

### Recruitment and eligibility criteria

The cross-sectional RESOURCE survey was designed to be completed by individuals with obesity in France, Germany, Italy, Spain, Sweden and the UK. Recruitment to the survey was managed by an existing consumer research panel, with a total reach of 3 million people across the six countries of interest. It was estimated that 20–40% of the separate panels from each country were living with obesity.

Adults (≥18 years old) with BMI ≥30 kg/m^2^ who reported interacting with primary or secondary healthcare services in the past 12 months were eligible for participation. Individuals who reported pregnancy during the past 12 months were excluded. The consumer research panel identified potentially eligible participants amongst those already enrolled in the panel, using the study inclusion criteria for guidance, and provided them with a unique online link to direct them to the survey. Recruitment was targeted to be nationally representative in terms of demographic factors, including household income bracket. Participants provided informed consent and answered screening questions to confirm their eligibility before completing the survey. Data were collected between May and June 2021.

The target sample size for survey participants was 1850; pre-specified target numbers per country were as follows: 100 participants from Sweden, 250 each from France and Germany, 300 from Italy, 450 from Spain and 500 from the UK. Once these targets had been reached, the survey closed, and could not be completed by further participants.

### Survey design and quality control

The survey was designed collaboratively by Novo Nordisk and Adelphi Real World, and translated into participants’ local languages. Participants were provided with the materials and instructions via a unique survey link, and were given a survey number to ensure anonymity. All participants were asked to provide informed consent before they could continue with the survey. The survey took ~20 min to complete and was completed only once per respondent. Upon completion, participants received a points-based e-reward with an equivalent cash value of £1. Quality control steps were put in place to ensure data integrity (see Supplementary Methods).

### Data collected

All survey data were self-reported. Participants answered screening questions to confirm that they were ≥18 years of age, lived in one of the countries of interest, had current weight and height measurements indicating a BMI of ≥30 kg/m^2^, had interacted with healthcare services in the past 12 months and had not been pregnant in this time. Eligible participants then completed questions in various categories, including demographic and clinical characteristics (see Supplementary Table S1 for a full list of variables collected).

Participants were asked to report any conditions for which they had received a diagnosis or were currently being treated, by selecting a specific condition or group of conditions from a list (Supplementary Table S2), which also included an option “Other conditions not listed above”. Some of the ~50 comorbidities collected in the survey have been examined in previous studies of obesity [7, 12, 13], and therefore their prevalence in the study population was of specific interest. These 15 conditions, termed ORCs in this study, were atherosclerotic cardiovascular disease (defined as experiencing at least one of coronary heart disease, peripheral artery disease, cerebrovascular disease and atherosclerosis), asthma, chronic kidney disease/kidney failure, dyslipidaemia, gastro-oesophageal reflux disease, heart failure, hypertension, musculoskeletal pain, obstructive sleep apnoea, osteoarthritis, polycystic ovary syndrome, prediabetes, psoriasis, T2D and urinary incontinence.

Questions on HCRU included treatments for weight management/reduction or current conditions, general practitioner visits and treatments administered, inpatient admissions, outpatient appointments and surgical procedures. Questions on weight loss strategies attempted in the past 12 months, whether prescribed by a healthcare professional or not, were also included in the survey.

Weight change in the past 12 months was calculated as the difference between each participant’s estimated weight 12 months ago and their estimated current weight. Clinically meaningful weight change was defined as a change of ≥5% in body weight. More stringent thresholds of a change of ≥10% or ≥15% in body weight were also analysed.

### Subgroups

For analysis of ORCs and HCRU, participants were grouped by obesity class (class I: BMI 30 to <35 kg/m^2^; class II: BMI 35 to <40 kg/m^2^; class III: BMI 40 to <70 kg/m^2^) and by number of ORCs. Weight loss strategies were assessed by obesity class, and estimated weight changes were assessed by specific weight loss strategies attempted, number of weight loss strategies attempted and obesity class.

### Analyses and handling of missing data

Descriptive statistics were used. Numeric variables are summarized by the mean, standard deviation (SD) and 95% confidence interval (CI). Categorical variables are summarized by the number and proportion of subjects in each category. Odds ratios (ORs) were calculated for the risk of hospitalization and risk of surgery by number of ORCs, using a logistic regression model adjusted for age and BMI.

To avoid missing data, surveys were programmed so that participants could not move forward to the next question without fully completing the current question. All questions needed to be completed for the survey to be finished (see Supplementary Methods). For categorical variables, “don’t know” responses were considered valid responses. For continuous variables, respondents were asked to estimate responses.

### Ethics

The study was performed in compliance with the European Pharmaceutical Market Research Association code of conduct. Fully de-identified respondent information was collated and aggregated. The study protocol was submitted to a centralized Institutional Review Board for methodological review, and received an approval from the Institutional Review Board commission on 15 March 2021.

## Results

### Study population and demographic characteristics

In total, 25,686 people were invited to take part in the survey. Of the 1850 eligible individuals who completed the survey, 1042 participants (56.3%) were in obesity class I, 496 (26.8%) in obesity class II and 312 (16.9%) in obesity class III. The mean age was 52.9 years (SD: 13.9) and 963 participants were women (52.1%). Most participants were Caucasian (*n* = 1665; 90.0%) and the majority (*n* = 1242; 67.1%) reported obtaining treatment via their country’s national health service. Table 1 shows demographic characteristics for the whole population, by obesity class and by number of ORCs.

**Table 1:** Demographic characteristics for participants in the RESOURCE survey.

	Total (*N* = 1850)	Obesity class I (*n* = 1042)	Obesity class II (*n* = 496)	Obesity class III (*n* = 312)	0 ORCs (*n* = 476)	1 ORC (*n* = 526)	2 ORCs (*n* = 362)	≥3 ORCs (*n* = 486)
*Age, years, mean (SD)*	52.9 (13.9)	53.3 (14.4)	52.6 (13.6)	51.8 (12.8)	45.6 (13.0)	51.5 (13.7)	55.7 (13.6)	59.3 (11.4)
*Women,* *n* (%)	963 (52.1)	485 (46.5)	279 (56.2)	199 (63.8)	288 (60.5)	285 (54.2)	168 (46.4)	222 (45.7)
*Race/ethnicity*, *n* (%)								
Caucasian	1665 (90.0)	943 (90.5)	446 (89.9)	276 (88.5)	422 (88.7)	472 (89.7)	323 (89.2)	448 (92.2)
Hispanic	42 (2.3)	23 (2.2)	14 (2.8)	5 (1.6)	7 (1.5)	13 (2.5)	12 (3.3)	10 (2.1)
Black/Afro-Caribbean	15 (0.8)	9 (0.9)	1 (0.2)	5 (1.6)	9 (1.9)	1 (0.2)	2 (0.6)	3 (0.6)
Asian	7 (0.4)	4 (0.4)	2 (0.4)	1 (0.3)	0 (0.0)	1 (0.2)	1 (0.3)	5 (1.0)
Other	121 (6.5)	63 (6.0)	33 (6.7)	25 (8.0)	38 (8.0)	39 (7.4)	24 (6.6)	20 (4.1)
*Insurance type,* *n* (%)								
National health service	1242 (67.1)	685 (65.7)	341 (68.8)	216 (69.2)	318 (66.8)	334 (63.5)	253 (69.9)	337 (69.3)
Private (self-paid)	271 (14.6)	170 (16.3)	62 (12.5)	39 (12.5)	68 (14.3)	75 (14.3)	50 (13.8)	78 (16.0)
Private insurance, covered	193 (10.4)	112 (10.7)	52 (10.5)	29 (9.3)	49 (10.3)	71 (13.5)	31 (8.6)	42 (8.6)
Other	72 (3.9)	39 (3.7)	20 (4.0)	13 (4.2)	18 (3.8)	26 (4.9)	15 (4.1)	13 (2.7)
Don’t know	72 (3.9)	36 (3.5)	21 (4.2)	15 (4.8)	23 (4.8)	20 (3.8)	13 (3.6)	16 (3.3)
*Number of ORCs,* *n* (%)								
0 ORCs	476 (25.7)	291 (27.9)	121 (24.4)	64 (20.5)	-	-	-	-
1 ORC	526 (28.4)	303 (29.1)	145 (29.2)	78 (25.0)	-	-	-	-
2 ORCs	362 (19.6)	206 (19.8)	100 (20.2)	56 (17.9)	-	-	-	-
≥3 ORCs	486 (26.3)	242 (23.2)	130 (26.2)	114 (36.5)	-	-	-	-

*ORC* obesity-related complication, *SD* standard deviation.

Demographic characteristics were generally similar across obesity classes, although there was a greater proportion of women in the higher compared with the lower obesity classes (class I: 46.5%; class II: 56.2%; class III: 63.8%). Compared with those who had fewer or no ORCs, participants with an increasing number of ORCs were likely to be older: mean age for those with no ORCs was 45.6 years (SD: 13.0), compared with 59.3 years (11.4) for those with ≥3 ORCs. Participants with more ORCs were also more likely to be men (0 ORCs: 60.5% women; 1 ORC: 54.2%; 2 ORCs: 46.4%; 3 ORCs: 45.7%). Demographic and clinical data by country are provided in Supplementary Table S3.

### Distribution of ORCs

Nearly 75% of participants had at least one of the 15 ORCs assessed in this study. In total, 526 (28.4%) had 1 ORC, 362 (19.6%) had 2 ORCs and 486 (26.3%) had ≥3 ORCs; 476 participants (25.7%) reported no ORCs (Table 1). When all of the approximately 50 comorbidities listed in the survey were accounted for, 132 participants (7.1%) reported no comorbidities at all. Higher obesity classes tended to have more ORCs: 242 participants with class I obesity (23.2%) had ≥3 ORCs, compared with 130 participants with class II obesity (26.2%) and 114 with class III obesity (36.5%). Supplementary Table S3 includes data on number of ORCs by country.

**Fig. 1:**
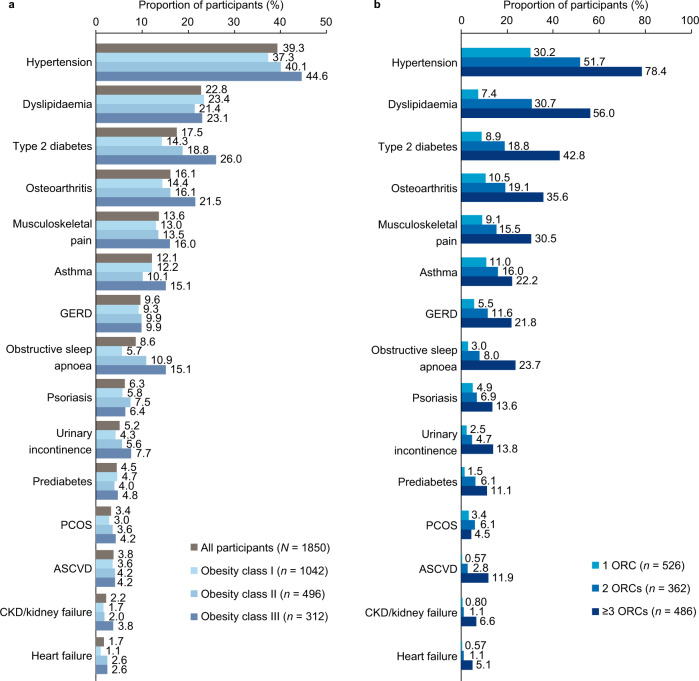
ORCs reported in the RESOURCE survey. **a** obesity class and **b** number of ORCs. ASCVD, atherosclerotic cardiovascular disease; CKD, chronic kidney disease; GERD, gastro-oesophageal reflux disease; ORC, obesity-related complication; PCOS, polycystic ovary syndrome.

The most frequent ORC was hypertension, which was reported by 39.3% of all participants (*n* = 727; Fig. 1a). Other frequently reported ORCs were dyslipidaemia (*n* = 422; 22.8%), T2D (*n* = 323; 17.5%) and osteoarthritis (*n* = 297; 16.1%). Some ORCs were reported by more participants in higher rather than lower obesity classes. Rates of obstructive sleep apnoea were >2.5 times higher for obesity class III than for class I, rates of T2D were nearly double and rates of osteoarthritis were ~1.5 times higher. Rates of hypertension, musculoskeletal pain and urinary incontinence were also notably higher for each increasing obesity class (Fig. 1a). In general, the ORCs most frequently reported by all participants were also the most frequent in participants with multiple ORCs. For example, 51.7% of participants with 2 ORCs (*n* = 187) and 78.4% of those with ≥3 ORCs (*n* = 381) had hypertension, and these proportions were 30.7% (*n* = 111) and 56.0% (*n* = 272) for dyslipidaemia (Fig. 1b). Supplementary Table S4 shows the numbers of participants reporting each ORC, by obesity class and number of ORCs.

### Healthcare resource use

In the preceding 12 months, 334 survey participants (18.1%) had experienced at least one inpatient admission (hospitalization), for any reason. Similar proportions of obesity classes I, II and III had experienced hospitalization (19.4%, 15.7% and 17.3%, respectively; Supplementary Table S5). In contrast, participants with more ORCs reported higher rates of hospitalization than those with fewer or no ORCs. Overall, 13.4% of participants with no ORCs had been hospitalized in the past year, compared with 15.4% of those with 1 ORC, 16.3% of those with 2 ORCs and 26.7% of those with ≥3 ORCs (Supplementary Table S5). Participants with any number of ORCs were significantly more likely to report hospitalization than those with no ORCs (Table 2); those with ≥2 ORCs were more than twice as likely to have experienced hospitalization (OR: 2.18 [95% CI: 1.57–3.06]; *P* < 0.001) and those with ≥3 ORCs were ~3 times more likely to have experienced hospitalization (OR: 2.98 [95% CI: 2.08–4.30]; *P* < 0.001). Of the survey participants who reported an inpatient admission, most reported that the admission had been preceded by a visit to the emergency department (*n* = 207; 62.0%).

**Table 2:** ORs for risk of reporting hospitalization or surgery, by number of ORCs.

Number of ORCs	Hospitalization		Surgery	
	OR *vs* 0 ORCs (95% CI)	*P-*value	OR vs 0 ORCs (95% CI)	*P-*value
≥1	1.73 (1.28–2.37)	**<0.001**	1.30 (0.96–1.77)	0.10
≥2	2.18 (1.57–3.06)	**<0.001**	1.45 (1.05–2.03)	**0.027**
≥3	2.98 (2.08–4.30)	**<0.001**	1.87 (1.31–2.69)	**<0.001**

Bold text indicates significant differences *versus* the group with no ORCs.

*CI* confidence interval, *OR* odds ratio, *ORC* obesity-related complication.

For all survey participants, the mean number of nights spent in hospital for any reason in the past 12 months was 1.3 (SD: 6.9; *n* = 1838). There appeared to be no relationship between obesity class and mean (SD) number of nights spent in hospital (1.4 [7.8]; 0.8 [4.4]; 1.6 [7.3] for the three classes, respectively), but participants with more ORCs had spent more nights in hospital than those with fewer or none (0 ORCs: 0.6 [3.1]; 1 ORC: 0.8 [4.4]; 2 ORCs: 1.7 [11.1]; ≥3 ORCs: 2.0 [7.7]).

In the preceding 12 months, 319 participants (17.2%) had undergone any surgical procedure, and these proportions were similar across obesity classes I, II and III (17.6%, 17.5% and 15.7%, respectively; Supplementary Table S5). In the group with no ORCs, 14.1% of participants reported a surgical procedure, compared with 15.8% of those with 1 ORC, 14.6% of those with 2 ORCs and 23.9% of those with ≥3 ORCs (Supplementary Table S5). Compared with participants who had no ORCs, participants with ≥2 ORCs (OR: 1.45 [95% CI: 1.05–2.03]; *P* = 0.027) or ≥3 ORCs (OR: 1.87 [95% CI: 1.31–2.69]; *P* < 0.001) were significantly more likely to report undergoing a surgical procedure (Table 2).

Most participants had received at least one prescription treatment in the past 12 months (*n* = 1478; 79.9%). This was driven largely by participants with ORCs: a minority of participants with no ORCs had been prescribed a treatment in the past 12 months (43.7%), but rates were much higher for those with at least one ORC (1 ORC: 86.5%; 2 ORCs: 93.9%; ≥3 ORCs: 97.7%).

Participants were asked if they had received a treatment administered by a healthcare professional in a healthcare setting, such as treatments administered via injection or intravenous drip, radiotherapy or physiotherapy. Overall, 596 participants (32.2%) had received at least one such treatment in the past 12 months. This proportion increased slightly with obesity class (class I: 29.8%; class II: 34.5%; class III: 36.5%), but had a stronger relationship with number of ORCs (0 ORCs: 18.7%; 1 ORC: 32.1%; 2 ORCs: 34.8%; ≥3 ORCs: 43.6%).

### Weight loss strategies

Overall, 1050 participants (56.8%) reported that they had received a diagnosis of overweight or obesity, or were currently receiving treatment for weight loss or management. A minority of these participants (*n* = 161; 15.3%) reported receiving a prescription medication for weight management, the majority of whom had received this from a general practitioner (*n* = 95; 59.0%) or a dietician (*n* = 57; 35.4%) in a primary care setting (*n* = 93; 57.8%) or a weight management clinic (*n* = 54; 33.5%).

**Fig. 2:**
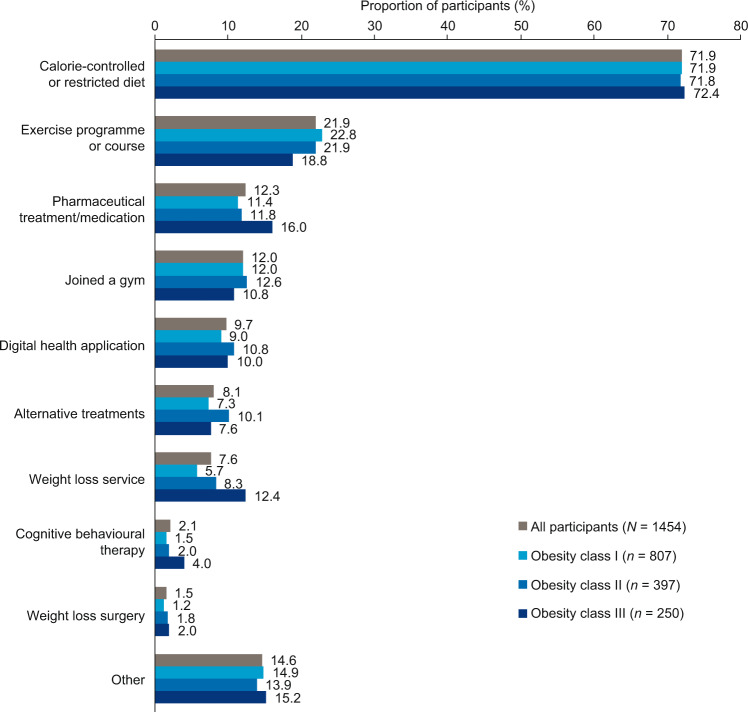
Weight loss strategies reported by survey participants. Digital health application refers to an application specifically for weight management. Alternative treatments include dietary supplements or herbal products. Weight loss service refers to both commercial services and programmes provided by the national health service.

The majority of participants (*n* = 1454; 78.6%) reported that they had attempted to lose weight in the past year. This proportion was similar for obesity classes I (*n* = 807; 77.4%), II (*n* = 397; 80.0%) and III (*n* = 250; 80.1%). Most participants had attempted only one strategy (*n* = 851; 58.5%). The most frequently reported weight loss strategy was a calorie-controlled or restricted diet (*n* = 1046; 71.9% of participants; Fig. 2 and Supplementary Table S6), and the second most frequent was an exercise programme or course, reported by a much smaller proportion of participants (*n* = 318; 21.9%). Overall, 81% of those who had attempted to lose weight used diet and/or exercise strategies. The distribution of weight loss strategies was similar across obesity classes. Use of pharmaceutical treatment or medication was reported most frequently in obesity class III (class I: 11.4%; class II: 11.8%; class III: 16.0%), as was use of a weight loss service (5.7%, 8.3% and 12.4%, respectively); however, overall rates of these strategies in the study population were low, at 12.3% and 7.6%, respectively.

### Weight loss

Overall, data on estimated weight changes during the past 12 months were available for 1753 participants (94.8%), and of the 1454 participants who reported attempting weight loss via one or more strategies, weight change data were available for 1383 people (95.1%). Overall, 73.4% of the latter group did not experience clinically meaningful weight loss of ≥5% (weight remained the same: *n* = 700 [50.6%]; weight increased: *n* = 316 [22.8%]). When a more stringent threshold of ≥10% weight loss was applied, 89.0% of those who attempted weight loss did not experience a clinically meaningful loss (weight remained the same: *n* = 1077 [77.9%]; weight increased: *n* = 154 [11.1%]). For a threshold of ≥15% weight loss, 95.1% did not experience a clinically meaningful loss (weight remained the same: *n* = 1239 [89.6%]; weight increased: *n* = 76 [5.5%]).

**Fig. 3:**
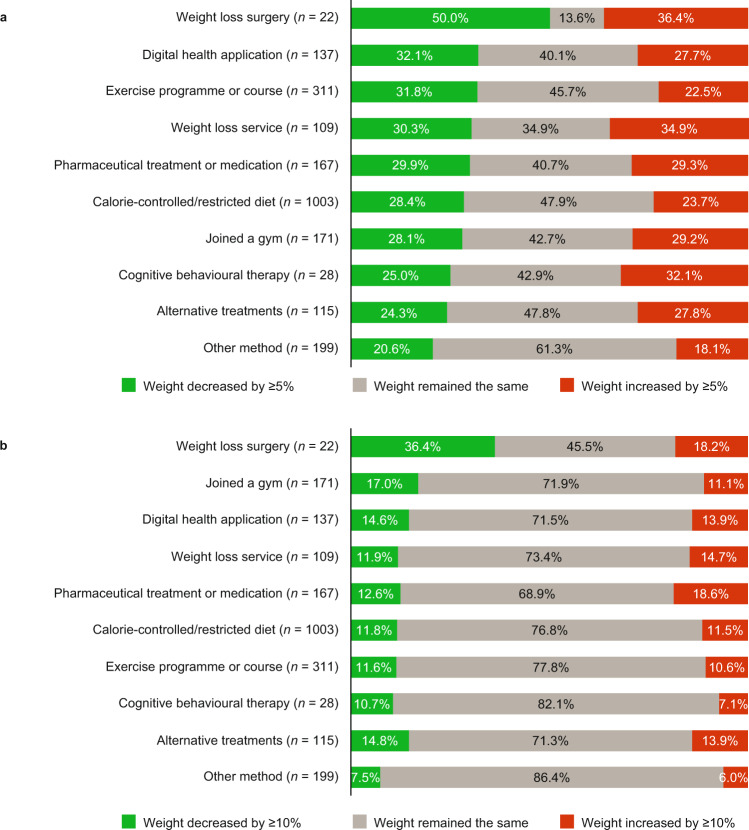
Estimated proportions of participants reporting use of a weight loss strategy who achieved clinically meaningful weight loss. **a** ≥5% and **b** ≥10%. Reported weight loss strategies are not mutually exclusive.

Weight loss of ≥5% was most frequently reported by participants who had undergone surgery (50.0% lost weight) or used a digital health application (32.1% lost weight; Fig. 3a). Weight loss of ≥10% was reported by 36.4% of participants who had undergone surgery, but by ≤17% of those who used other strategies (Fig. 3b). Regardless of the number of weight loss strategies attempted, most participants did not experience clinically meaningful weight loss of ≥5%. Participants who had attempted two or more different strategies were more likely to achieve weight loss of ≥5% than those who had attempted only one strategy (33.1% *vs* 22.4% reported weight loss), and those who attempted three or more strategies were most likely to achieve ≥10% weight loss or ≥15% weight loss (Table 3). Weight loss rates were similar across the three obesity classes: 26.7%, 26.9% and 25.3% in each class, respectively, reported ≥5% weight loss. Supplementary Table S7 shows weight loss data by country and obesity class.

**Table 3:** Percentages of participants achieving weight loss, by number of strategies attempted.

	Number of weight loss strategies attempted		
	1 (*n* = 851)	2 (*n* = 337)	≥3 (*n* = 195)
*Threshold for clinically meaningful weight loss:* *≥5%*			
Weight decreased, *n* (%)	191 (22.4)	110 (32.6)	66 (33.8)
Weight remained the same, *n* (%)	476 (55.9)	147 (43.6)	77 (39.5)
Weight increased, *n* (%)	184 (21.6)	80 (23.7)	52 (26.7)
*Threshold for clinically meaningful weight loss:* *≥10%*			
Weight decreased, *n* (%)	81 (9.5)	35 (10.4)	36 (18.5)
Weight remained the same, *n* (%)	675 (79.3)	265 (78.6)	137 (70.3)
Weight increased, *n* (%)	95 (11.2)	37 (11.0)	22 (11.3)
*Threshold for clinically meaningful weight loss:* *≥15%*			
Weight decreased, *n* (%)	38 (4.5)	12 (3.6)	18 (9.2)
Weight remained the same, *n* (%)	765 (89.9)	308 (91.4)	166 (85.1)
Weight increased, *n* (%)	48 (5.6)	17 (5.0)	11 (5.6)

## Discussion

The RESOURCE survey was designed to collect a broad range of self-reported healthcare information from people with obesity in six European countries. Firstly, we show that ORCs are frequently reported by people with obesity, particularly in obesity class III. Secondly, the presence of multiple ORCs is linked to a greater likelihood of hospitalization or other HCRU. Thirdly, although most people reported attempting to lose weight, the majority did not experience clinically meaningful weight loss of ≥5%.

In the RESOURCE survey, more than 25% of participants had ≥3 of the 15 ORCs considered to be strongly linked to obesity, and nearly 75% of participants had ≥1 ORC, the most frequently reported being hypertension, dyslipidaemia and T2D. Furthermore, the chance of having multiple ORCs was linked to obesity class, and some specific ORCs were considerably more frequent in individuals in the highest obesity class. Notably, participants in obesity class III were much more likely to report obstructive sleep apnoea or T2D than those in obesity class I. This finding aligns closely with the results of a previous UK study, in which sleep apnoea and T2D were found to be the ORCs with the greatest increase in risk associated with obesity class III, compared with normal weight [12]. It should also be noted that, rather than experiencing a combination of less serious conditions, most participants with multiple ORCs had one or more chronic conditions requiring continuous management. Of the participants who reported ≥3 ORCs, nearly 80% had hypertension, more than 50% had dyslipidaemia and more than 40% had T2D. These self-reported conditions are likely to represent diagnosed conditions, and therefore it is probable that additional participants had cardiovascular risk factors or prediabetes, but have not yet been diagnosed.

ORCs are known to be linked to healthcare costs in individuals with obesity [7], and the results of the survey indicate that increasing number of ORCs is a greater risk factor for higher HCRU than increasing obesity class. The likelihood of reporting hospitalization or surgery was similar across obesity classes, but participants with any number of ORCs had a significantly higher chance of reporting hospitalization than participants with no ORCs, and participants with multiple ORCs had a significantly higher chance of reporting any surgical procedure. The presence of ORCs was also linked to requirement for prescription medication: 44% of participants with no ORCs but 87% of those with one ORC had received at least one prescription treatment in the past year. This close link between ORCs and HCRU shows the need for a shift in the treatment perspective for obesity, by both clinicians and healthcare systems, from focusing only on change in body weight to also assessing the benefits for overall health status to be gained via weight management. This holistic view, which takes into account the reductions in comorbidities that may be gained via weight management, is one that recognizes unmet need and attempts to address the full clinical impact of obesity.

Our findings on weight loss strategies and estimated weight changes indicate that most people with obesity in Europe attempt to lose weight, but are unsuccessful. Most participants reported attempting weight loss via diet or exercise, but fewer than 30% experienced clinically meaningful weight loss of ≥5% during the past 12 months. Surgical intervention was the most successful strategy; however, as suggested by the small sample size for this group (*n* = 22; 1.2% of all participants), weight loss surgery is not indicated for all people with obesity, and may be unsuitable or not desired by many people who are eligible, with the risks of surgery and the lifestyle changes required acting as barriers to uptake [14]. Nearly half of participants in the survey had not received a formal diagnosis of overweight or obesity, and only 15% had been prescribed a treatment for weight management. Together, these data highlight a clear unmet need for support with weight loss, structured monitoring of weight loss strategies, and access to effective therapy. This would help to ease both the clinical and the economic impacts of obesity. In a study using UK data, weight loss of 13% was estimated to reduce the risks of multiple ORCs, including hypertension, dyslipidaemia and T2D [13], and our results suggest that limiting the development of these comorbidities would considerably reduce risks of hospitalization, surgery and other HCRU.

The key value of the RESOURCE survey is the breadth of data captured and the collection of evidence on weight loss strategies. The results indicate that healthcare professionals are not involved in participants’ weight management in many cases, suggesting that this information would not be available in other sources of real-world data, such as medical records. The relatively large sample size and geographic scope of this study mean that the results provide a broad overview of obesity in Western Europe. However, the possibility of selection bias in patient surveys, which are dependent on the ability to reach representative eligible individuals, must be acknowledged. Various considerations may influence whether people choose to participate in surveys, including factors relevant to obesity and HCRU, such as socioeconomic and employment status, age and physical health.

Although self-reporting of data is a necessary limitation of patient surveys, checks and quality control incorporated into the survey design were used to confirm data relevance and validity. There remains, however, the potential for inaccuracies and recall bias in the responses to the screening criteria, and in the survey results. The reliance on BMI alone as a measure of obesity means that individuals with high muscle mass rather than excess fat could have been included in the study sample. More importantly, weight change was based on comparison between a historical weight estimate and current weight, which could be either a measurement or an estimate. Timings of weight change, or fluctuations over the past 12 months, were not recorded, and could not be linked to the timings or duration of weight loss attempts, meaning that weight loss strategies may have been associated with some temporary weight loss, but followed by subsequent gain. Therefore, these results cannot be used to compare the likelihood of weight loss between particular strategies, but rather to demonstrate a need for improved support and future research. Structured, longitudinal studies tracking weight loss strategies are required to quantify weight change accurately; similarly, future studies should be designed to assess the relationship between development of ORCs and subsequent HCRU in greater detail.

## Conclusions

The results of the RESOURCE survey show that the majority of people living with obesity report that they have one or more ORCs, which are associated with increased HCRU. The chances of experiencing multiple ORCs increases with obesity class; however, the occurrence of ORCs and the associated increase in HCRU are common in all obesity classes. There is evidence that most people with obesity attempt to lose weight, but the majority do not achieve weight loss. Increased support to prevent the development and progression of obesity is likely to bring a range of clinical benefits in addition to reducing the effects of excess weight, and is needed to limit the impact of ORCs on healthcare spending.

## Supplementary information

Supplementary methods

Supplementary RESOURCE survey

Supplementary Table 1

Supplementary Table 2

Supplementary Table 3

Supplementary Table 4

Supplementary Table 5

Supplementary Table 6

Supplementary Table 7

Supplementary Information

## Data Availability

All data supporting the conclusions of these analyses are presented in the manuscript or the supplementary material. Details of additional data can be obtained from the study authors upon reasonable request.
