# Supplementary material for: Obesity-related complications, healthcare resource use and weight loss strategies in six European countries: the RESOURCE survey
Source: Int J Obes (Lond). 2023 May 31;47(8):750–7. doi: 10.1038/s41366-023-01325-1 (PMC10359184; doi:10.1038/s41366-023-01325-1)
Supplement: Supplementary file 1 — Supplementary methods [file 41366_2023_1325_MOESM1_ESM.docx]

## Supplementary methods

### Survey design, development and quality control

- Participants were provided with the telephone number of the consumer panel to request technical assistance.
- Quality control steps to ensure data integrity were as follows.
  - Logic was programmed into the survey to ensure that all applicable questions were completed by participants.
  - Normal ranges were also set as field parameters to ensure that the data entered were within expected ranges.
  - Initial checks were completed to ensure that the online survey was functioning as expected, and data checks were carried out throughout the survey period to identify and remove any respondents who took very short amounts of time to complete the survey or answered with the same response option, quickly, for many questions.
- Following programming into the online survey link, translation was carried out by a language services provider (CETRA). Survey links and content were then checked for functionality, accuracy and appropriateness by local language and country experts.
- For some questions in the survey, participants answered stem questions and then skipped certain questions depending on the answers they provided. For example, if they reported that they had not used a certain resource in the past 12 months, then subsequent questions on use of this resource were skipped.
- The full survey is included as a separate supplementary file.
