# Supplementary material for: Obesity-related complications, healthcare resource use and weight loss strategies in six European countries: the RESOURCE survey
Source: Int J Obes (Lond). 2023 May 31;47(8):750–7. doi: 10.1038/s41366-023-01325-1 (PMC10359184; doi:10.1038/s41366-023-01325-1)
Supplement: Supplementary file 2 — Supplementary RESOURCE survey [file 41366_2023_1325_MOESM2_ESM.docx]

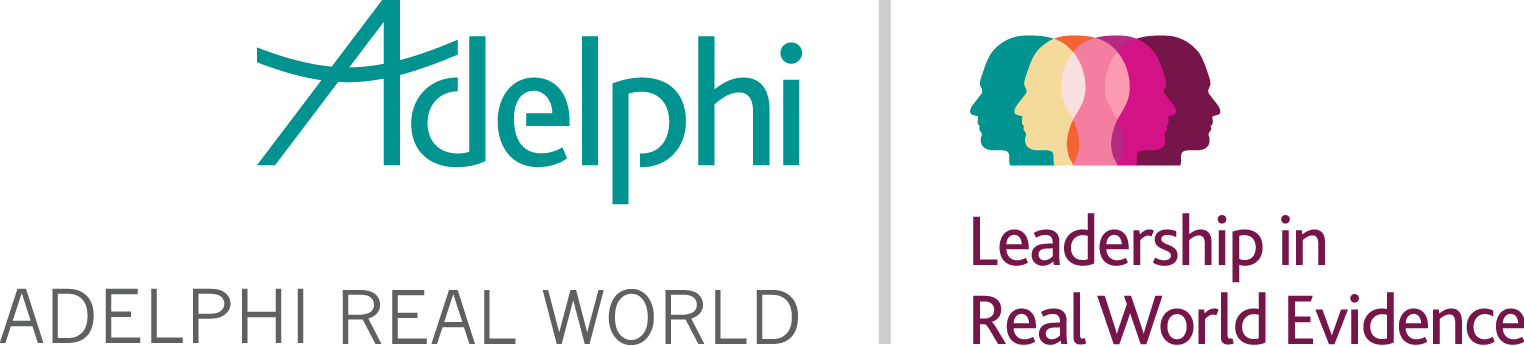

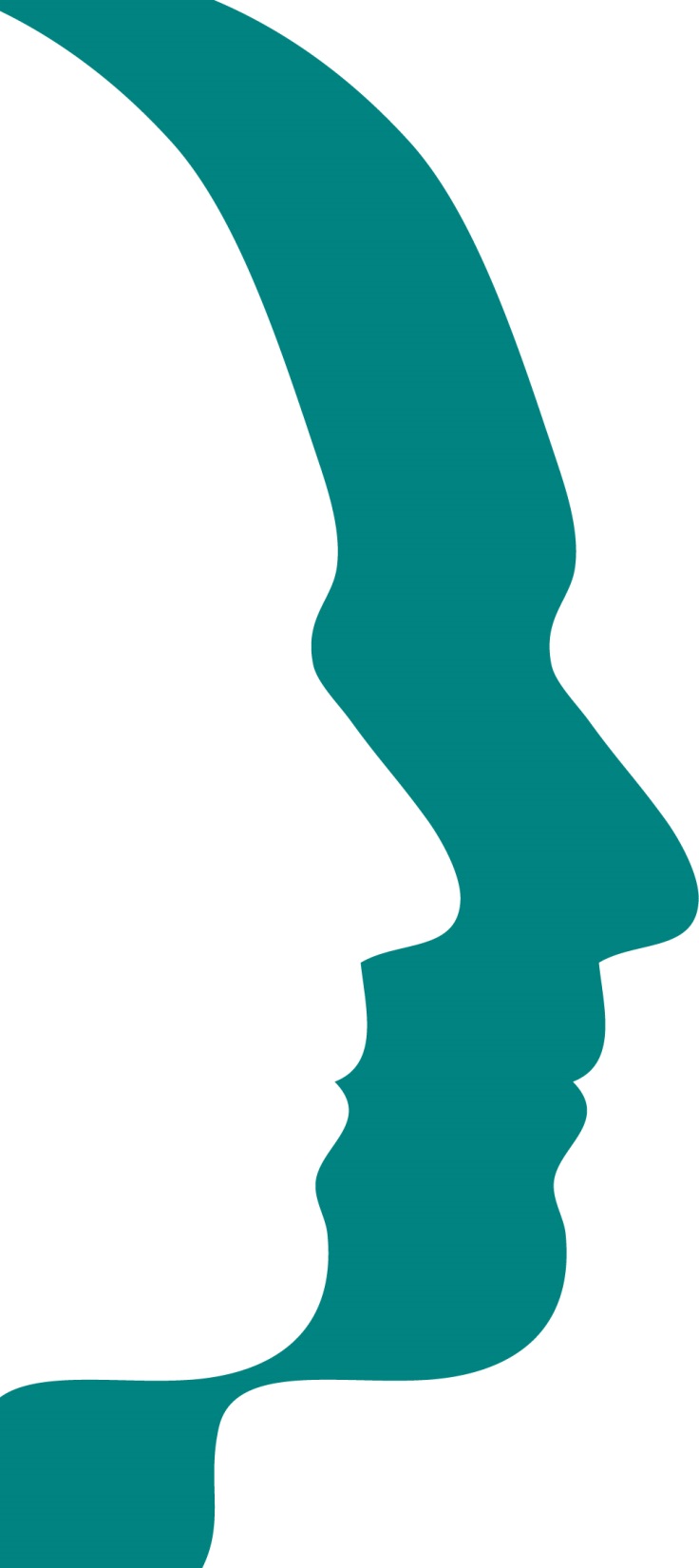
br

**RESearch survey assessing individuals with Obesity to Understand their healthcare Resource use and Characteristics within the EU5 and Sweden (RESOURCE)**

Novo Nordisk

V7.0

February 2021

**Survey Sections:**

The survey will cover the following areas:

[WELCOME PAGE AND DISCLAIMER 2](#_Toc63778907)

[SECTION A: PATIENT SCREENER 7](#_Toc63778908)

[SECTION B: PATIENT CHARACTERISTICS 9](#_Toc63778909)

[SECTION C: TREATMENT 16](#_Toc63778910)

[SECTION D: HEALTHCARE RESOURCE USE (LAST 12 MONTHS) 24](#_Toc63778911)

[SECTION E: COVID-19 36](#_Toc63778912)

END OF SECTION

WELCOME PAGE AND DISCLAIMER

Survey-related contact email: [dynata_hchelp@dynata.com](mailto:dynata_hchelp@dynata.com)

Thank you for agreeing to participate in this research survey conducted by Adelphi Real World on behalf of a pharmaceutical company. Adelphi Real World would like everybody to be completely honest with their views and everything you say will be treated in total confidence.

The purpose of this survey is to understand the characteristics, treatment history, and healthcare resource use of individuals with a Body Mass Index (BMI) greater than or equal to 30kg/m^2^ in Europe and the data will be used to inform strategies for the reimbursement of treatments.

The survey will last approximately 20 minutes. Upon completion of the survey, you will receive a points based e-reward that has an equivalent cash value of £1 as remuneration in appreciation of your time and cooperation. The consumer panel will have informed you about the payment details.

We will comply with all national laws protecting your personal data and with relevant guidelines including ESOMAR, EphMRA <Germany only ADM/ BVM> and all other relevant national codes of practice. The aim of this research is to gain your views and is not intended to be promotional and no one will try to sell you anything. All information provided will remain confidential and will only be reported to the commissioning client, combined with other respondent’s data so there is no information that can identify you. Your information may be aggregated and shared with individuals and organizations that watch over this research, including:

- People who work with the research sponsor
- Government agencies, such as the Food and Drug Administration
- The Institutional Review Board (IRB) that reviewed this research

We protect your information from disclosure to others to the extent required by law. We cannot promise complete secrecy, so there is a small risk of loss of confidentiality. There will be no direct benefit to you for participating, but your participation may help researchers and other patients in the future.

We ask you to review and agree to the following statements prior to participating in the survey.

- I confirm that I am happy to take part in this survey voluntarily and know that I may terminate the survey or withhold information if I so wish. I understand that I can withdraw my consent at any point. My alternative is not to participate, and my decision to not participate or to leave the survey will not result in any penalty or loss of benefits to which I am otherwise entitled.
- I understand that all data from this survey will only be used for research purposes.
- Adequate data protection measures will be put in place to comply with the Data Protection Laws of the country in which the survey takes place. For example, as determined by the: EU General Data Protection Legislation (GDPR); EU-US privacy shield <https://www.privacyshield.gov/list> .

Adverse Event Reporting and Consent

- We are required by our sponsor to pass on details of adverse events/product complaints for any of the sponsor’s products, mentioned during the course of this research survey. This will be solely for drug safety to fulfill their obligation to the regulatory authorities.
- If this happens, we will need to collect details and report the event.
- We will initially forward the adverse event report to the sponsor’s pharmacovigilance department anonymously. Any requests for further information from pharmacovigilance will come through the recruiting agency.

| 1 | ⭘ | I would like to proceed and give permission for the consumer panel to contact me to ask if I am willing to provide more information about an adverse event mentioned by me during the survey |
| --- | --- | --- |
| 2 | ⭘ | I would like to proceed but do NOT give permission for the consumer panel to contact me to request more information about an adverse event mentioned by me during the survey |
| 3 | ⭘ | I don’t want to proceed and want to end the survey here |

<IF SELECT 3 ‘I don’t want to…’ END THE SURVEY – SCREENOUT TEXT SHOULD READ “THANK YOU FOR CONSIDERING THIS RESEARCH, WE HOPE THAT YOU CONSIDER PARTICIPATING IN FUTURE SURVEYS”>

Institutional Review Board

This research is being overseen by an Institutional Review Board (‘IRB’). An IRB is a group of people who perform independent review of research surveys. You may talk to them at at 855-818-2289 or researchquestions@wcgirb.com if: :

- - You have questions, concerns, or complaints that are not being answered by the research team.
  - You are not getting answers from the research team.
  - You cannot reach the research team.
  - You want to talk to someone else about the research.
  - You have questions about your rights as a research subject.

Protocol No. NN9080

Your Rights Under Data Privacy Laws

Click here to view our statement regarding data protection, privacy, invisible processing and ways to contact us.

Contact Details

If you have questions, concerns, or complaints, or think this research has hurt you or made you sick, talk to the survey team (consumer panel) as noted below.

In case of further queries, to access, modify or delete your personal data or if you wish to withdraw your consent at any time please contact:

**Consumer panel**

Contact Consumer panel: [dynata_hchelp@dynata.com](mailto:dynata_hchelp@dynata.com)

Consumer panel’s privacy policy [www.dynata.com](http://www.dynata.com)

**Adelphi Real World**

Contact Adelphi Real World at: [arw-compliance@adelphigroup.com](mailto:arw-compliance@adelphigroup.com)
Find out more about Adelphi Real World at [www.adelphirealworld.com](http://www.adelphirealworld.com)
Read Adelphi Real World’s Privacy Policy at: <http://www.adelphigroup.com/privacypolicy.pdf>

By participating in the survey, you acknowledge and confirm you understand that all survey materials are proprietary to the survey owner and must not be used for any other purpose than participation in the survey or disclosed to any third party without the express written permission of the survey owner.

Consent

Select **YES** below to say you have read, understand and accept the statements contained in this “Consent to Participate” form and agree to take part in the survey on the basis of these statements**.**

| 1 | ⭘ | Yes |
| --- | --- | --- |
| 2 | ⭘ | No |

<IF SELECT 2 ‘NO’ END THE SURVEY – SCREENOUT TEXT SHOULD READ “THANK YOU FOR CONSIDERING THIS RESEARCH, WE HOPE THAT YOU CONSIDER PARTICIPATING IN FUTURE SURVEYS”>

Pop Up Privacy Statement

Adelphi Real World would like to thank you for taking part in this survey.

All information provided will remain confidential and will only be reported combined with other respondent’s data so there is no information which can identify you. We will not send you unsolicited mail or pass your personal data to a third party.

Your co-operation is voluntary at all times. No personal information is sought from or about you, without your prior knowledge and agreement. You are entitled at any stage of the survey, or subsequently, to ask that part or all of your survey record be destroyed or deleted. Wherever reasonable and practical we will carry out such a request.

We automatically capture information about your device, presence of Flash, operating system, web browser type, web browser version and screen resolution in order to deliver a survey best suited to your software. We do no other invisible processing of data from your computer.

Your survey responses are collected, stored or processed by our service providers, both within and outside the European Economic Area (EEA). They are contractually bound to keep any information confidential and must protect it with security standards and practices that are equivalent to our own.

**Your rights under Data Privacy Laws**

- The European Union’s General Data Protection Regulation (GDPR) and other countries’ data privacy laws provide certain rights for data subjects. This consent form and Adelphi’s privacy policy <http://www.adelphigroup.com/privacypolicy.pdf> provides information on how we collect and use (process) your personal data.
- In many countries, you have a right to access, modify or request deletion of your personal data and to lodge a complaint with the appropriate data protection authority if you have concerns about how your personal data is processed. Please see consumer panel’s privacy policy < [privacy@dynata.com](mailto:privacy@dynata.com)> and Adelphi’s privacy policy <http://www.adelphigroup.com/privacypolicy.pdf> for details on how to exercise these rights, or contact us using the contact details shown in the Contacts section.
- Adelphi Real World will not retain your personal data for longer than is necessary. At the end of the research project Adelphi Real World will redact (remove) the personal data no longer required for the project. This means that most of the personal data collected during the research project is deleted and there is minimal personal data retained by Adelphi Real World. The consumer panel may hold personal data about you as part of the management of their database or panel. Please consult the consumer panel’s privacy policy, their terms and conditions, or contact them directly, for further details of the personal data they hold.

**TO CONTACT US – ALL COUNTRIES**

In case of further queries, to access, modify or delete your personal data or if you wish to withdraw your consent at any time, please contact the survey team (fieldwork agency) who enrolled you:

**Consumer panel**

Contact consumer panel: **www.dynata.com**

Read consumer panel’s privacy policy: privacy@dynata.com

**Adelphi Real World**

You can contact Adelphi Real World at: [arw-compliance@adelphigroup.com](mailto:arw-compliance@adelphigroup.com)

You can find out more about Adelphi Real World at [www.adelphirealworld.com](http://www.adelphirealworld.com)

You can read Adelphi Real World’s Privacy Policy here: <http://www.adelphigroup.com/privacypolicy.pdf>

SECTION A: PATIENT SCREENER

**The screener aims to identify whether you are a suitable candidate for participation in this survey**.

AQ1. Are you 18 years or older? <ASK ALL>

**Please select one answer.**

< SINGLE CODE>

| 1 | ⭘ | Yes |
| --- | --- | --- |
| 2 | ⭘ | No |
| 3 | ⭘ | Don’t know |

**<SCREEN OUT IF code ‘2’ or ‘3’ selected>**

AQ2a. In which country do you currently reside? <ASK ALL>

**Please select one answer.**

<SINGLE CODE>

| 1 | ⭘ | France |
| --- | --- | --- |
| 2 | ⭘ | Germany |
| 3 | ⭘ | Italy |
| 4 | ⭘ | Spain |
| 5 | ⭘ | Sweden |
| 6 | ⭘ | United Kingdom |

**<SCREEN OUT IF COUNTRY OTHER THAN LOCATION WHERE PATIENT RECRUITED FROM IS SELECTED>**

AQ3. Please enter your estimated current weight in Kg **[OR** Stones/Lbs **[SHOW IN UK ONLY]** <ASK ALL>

<NUMERIC & SINGLE CODE>

| ________________ | Kg <RANGE: 1-500> |  |  |
| --- | --- | --- | --- |
| ________________ | Stones <RANGE: 1-99> | ________________ | Lbs <RANGE: 0-13> |
| ⭘ | Prefer not to say |  |  |

**<’Kg’, ‘Stones/Lbs’, and ‘Prefer not to say’ are MUTUALLY EXCLUSIVE, show error message if values entered for both ‘*’Please provide a response in ‘Kg’, <OR ’Stones/Lbs’ <<UK only>>>, OR ‘prefer not to say*’>**

**<Show ‘Kg’ for all countries, only show Stones/Lbs for UK; adjust wording of question appropriately depending on country>**

**<’Prefer not to say’ MUTUALLY EXCLUSIVE to numeric options>**

**<Prompt out of range error message: ‘’*Your response is not within the range we expected. If this response is correct, select ‘next’ to confirm, otherwise amend your response*’’>**

**<Screen out if ‘Prefer not to say’ selected>**

AQ4. Please enter your estimated current height **[SHOW IN UK ONLY:** in feet & inches **OR**] cm <ASK ALL>

<NUMERIC>

| ________________ | Feet <RANGE 1-9> | ________________ | Inches <RANGE: 0-11> |
| --- | --- | --- | --- |
| ________________ | Cm <RANGE: 1-299> |  |  |

**<FEET & INCHES ARE MUTUALLY EXCLUSIVE TO Cm, SHOW ERROR MESSAGE IF VALUES ENTERED FOR BOTH ‘*’PLEASE PROVIDE A RESPONSE FOR FEET & INCHES OR CM ONLY’’*>**

**<Show ‘cm’ for all countries, feet and inches to show for UK only; adjust wording of question appropriately depending on country>**

**<Prompt Out of Range Error Message: ‘*Your response is not within the range we expected. If this response is correct, select ‘next’ to confirm, otherwise amend your response’*>**

**<ALLOW ENTRY TO 1 DP>**

**<Program a new variable for BMI: (weight (lb) / [height (in))]2 x 703 OR for metric units, weight (kg)/ [height (m)]2 (where height in m= height in cm/100)>**

**<SCREEN OUT IF BMI CALCULATED IS <30 >**

AQ5. Have you been pregnant at any time in the past 12 months? <ASK ALL>

**Please select one answer.**

< SINGLE CODE>

| 1 | ⭘ | Yes |
| --- | --- | --- |
| 2 | ⭘ | No |
| 3 | ⭘ | Don’t know |

**<SCREEN OUT IF CODE 2 (NO) IS NOT SELECTED>**

AQ6. Have you had any interaction, for any condition, with primary care (including general practitioners, pharmacists etc.) or secondary care (e.g. hospital or specialist health care services) in the last 12 months? <ASK ALL>

**Please select one answer.**

< SINGLE CODE>

| 1 | ⭘ | Yes |
| --- | --- | --- |
| 2 | ⭘ | No |
| 3 | ⭘ | Don’t know |

**<SCREEN OUT IF CODE 1 (YES) IS NOT SELECTED**

SECTION B: PATIENT CHARACTERISTICS

**This section aims to collect basic information about you.**

BQ1. What is your biological sex? <ASK ALL>

**Please select one answer.**

<SINGLE CODE>

| 1 | ⭘ | Male |
| --- | --- | --- |
| 2 | ⭘ | Female |
| 3 | ⭘ | Prefer not to say |

BQ2. What is your age? <ASK ALL>

<NUMERICAL & SINGLE CODE>

| ______________ | Years <Range: 18-89> | OR | ⭘ | 90 years or above |
| --- | --- | --- | --- | --- |

BQ3. What is your ethnic group? <ASK ALL>

**Please select one answer.**

<SINGLE CODE>

| 1 | ⭘ | Caucasian |
| --- | --- | --- |
| 2 | ⭘ | Hispanic |
| 3 | ⭘ | Black / Afro-Caribbean |
| 4 | ⭘ | Asian |
| 5 | ⭘ | Other |

BQ4a. Do you currently have healthcare insurance? <ASK ALL>

*Please state yes if your healthcare is covered by a national health service*

**Please select one answer.**

<SINGLE CODE>

| 1 | ⭘ | Yes |
| --- | --- | --- |
| 2 | ⭘ | No |

<SHOW TO PATIENTS SELECTING CODE 1 ‘YES’ AT BQ4a>

BQ4b. What is the name of your plan/coverage option/insurance type?

| **FRANCE** | **GERMANY** | **ITALY** | **SPAIN** | **SWEDEN** | **UK** |
| --- | --- | --- | --- | --- | --- |
| Protection Universelle Maladie (PUMa) | Gesetzliche Krankenversicherung | Servizio Sanitario Nazionale | Sistema Nacional de Salud (SNS) | National Healthcare system / Universal healthcare coverage | National Health Service (NHS) |
| ⭘ | ⭘ | ⭘ | ⭘ | ⭘ | ⭘ |
| PUMa + CMU-C, mutuelle or assurance privée | Private Krankenversicherung | Servizio Sanitario Nazionale e Assicurazione sanitaria privata | Seguro médico privado | Complimentary private voluntary health insurance | Private self-paid |
| ⭘ | ⭘ | ⭘ | ⭘ | ⭘ | ⭘ |
| Pas de remboursement par l’assurance | Gesetzliche mit privater Zusatzversicherung | Assicurazione sanitaria privata | Sistema Nacional de Salud (SNS), y seguro médico privado |  | Private, insurance covered |
| ⭘ | ⭘ | ⭘ | ⭘ |  | ⭘ |
| Other | Other | Other | Other | Other | Other |
| ⭘ | ⭘ | ⭘ | ⭘ | ⭘ | ⭘ |
| Don’t know | Don’t know | Don’t know | Don’t know | Don’t know | Don’t know |
| ⭘ | ⭘ | ⭘ | ⭘ | ⭘ | ⭘ |

< Only show FRANCE options for participants residing in France; only show GERMANY options for participants residing in Germany; only show ITALY options for participants residing in Italy; only show SPAIN options for participants residing in Spain; only show SWEDEN options for participants residing in Sweden; only show UK options for participants residing in the UK>

BQ5. What is your current smoking status? <ASK ALL>

<SINGLE CODE>

**Please select one answer.**

| 1 | ⭘ | Current Smoker |
| --- | --- | --- |
| 2 | ⭘ | Ex-smoker |
| 3 | ⭘ | Non-smoker (never smoked) |

BQ6a. Have you been told by a healthcare practitioner that you have been diagnosed with any of the following conditions or are you currently being treated for any of the following conditions? <ASK ALL>

**Please select all that apply.**

<MULTICODE>

|  |  | **Weight** |
| --- | --- | --- |
| 1 | 🞎 | Weight Management/Reduction (Overweight or Obesity) |
|  |  | **Rheumatic diseases** |
| 2 | 🞎 | Osteoarthritis (OA) (a condition causing joint pain and stiffness) |
| 3 | 🞎 | Musculoskeletal pain (pain that affects the muscles, bones, ligaments, tendons and nerves) |
| 4 | 🞎 | Rheumatoid Arthritis |
|  |  | **Metabolic conditions** |
| 5 | 🞎 | Type 1 Diabetes |
| 6 | 🞎 | Type 2 Diabetes (T2DM) |
| 7 | 🞎 | Prediabetes (High blood sugar but not yet enough to be considered type 2 diabetes) |
| 8 | 🞎 | High cholesterol (Dyslipidemia) |
| 9 | 🞎 | Chronic Kidney Disease |
| 10 | 🞎 | Liver/Gallbladder/Pancreatic Disease – Non-alcoholic steatohepatitis (NASH)/Non-alcoholic fatty liver disease (NAFLD) |
| 11 | 🞎 | Kidney failure/Dialysis |
| 12 | 🞎 | Thyroid Disease |
|  |  | **Respiratory conditions** |
| 13 | 🞎 | Asthma |
| 14 | 🞎 | Chronic obstructive pulmonary disease (COPD) (lung conditions causing breathing difficulties e.g. emphysema, chronic bronchitis) |
| 15 | 🞎 | Obstructive sleep apnea (OSA) |
|  |  | **Cardiovascular conditions** |
| 16 | 🞎 | High blood pressure (Hypertension) |
| 17 | 🞎 | Hypertensive heart disease |
| 18 | 🞎 | Cerebrovascular Disease (Group of conditions, diseases and disorders that affect blood vessels and blood supply to the brain) |
| 19 | 🞎 | Coronary Heart Disease (Ischemic heart disease) |
| 20 | 🞎 | Heart failure |
| 21 | 🞎 | Cardiomyopathies (Diseases that affect the heart’s ability to pump blood around the body) |
| 22 | 🞎 | Deep vein thrombosis and pulmonary embolism (DVT and PE) |
| 23 | 🞎 | Cardiac arrest |
| 24 | 🞎 | Peripheral artery disease (Build up of fatty deposits in the arteries restricts blood supply to leg muscles) |
| 25 | 🞎 | Atrial fibrillation and flutter |
| 26 | 🞎 | Atherosclerosis (Arteries clogged with fatty substances) |
| 27 | 🞎 | I have a cardiovascular condition, but I am unsure which one |
|  |  | **Cancers** |
| 28 | 🞎 | Meningioma (A tumour that forms on the membranes that surround the brain and spinal cord) |
| 29 | 🞎 | Multiple myeloma |
| 30 | 🞎 | Esophageal cancer (cancer of the esophagus) |
| 31 | 🞎 | Cancers of the thyroid |
| 32 | 🞎 | Breast cancer |
| 33 | 🞎 | Gallbladder cancer |
| 34 | 🞎 | Stomach cancer |
| 35 | 🞎 | Liver cancer |
| 36 | 🞎 | Pancreatic cancer |
| 37 | 🞎 | Kidney cancer |
| 38 | 🞎 | Ovarian cancer |
| 39 | 🞎 | Uterine cancer |
| 40 | 🞎 | Colorectal cancer |
| 41 | 🞎 | Non-melanoma skin cancer |
| 42 | 🞎 | Other cancer |
|  |  | **Other conditions** |
| 43 | 🞎 | PCOS (polycystic ovary syndrome) |
| 44 | 🞎 | Urinary incontinence |
| 45 | 🞎 | Psoriasis |
| 46 | 🞎 | Gastroesophageal reflux disease (GERD) (acid reflux) |
| 47 | 🞎 | Depression |
| 48 | 🞎 | Stomach ulcers(peptic ulcers) |
| 49 | 🞎 | Liver failure |
| 50 | 🞎 | Other condition(s) not listed above |
| 51 | ⭘ | None of the above <mutually exclusive with codes 1-50> |

Codes 5 , 6 and 7 are mutually exclusive – only one can be selected

BQ6b. When were you first diagnosed with or treated for each of these conditions?

<Show conditions selected in BQ6a>

<SINGLE CODE PER ROW>

**Please select one answer.**

|  |  | **Less than 6 months ago** | **6-12 months ago** | **More than 12 months ago** |
| --- | --- | --- | --- | --- |
|  | **Weight** |  |  |  |
| 1 | Weight Management/Reduction (Overweight or Obesity) | ⭘ | ⭘ | ⭘ |
|  | **Rheumatic diseases** |  |  |  |
| 2 | Osteoarthritis (OA) | ⭘ | ⭘ | ⭘ |
| 3 | Musculoskeletal pain (pain that affects the muscles, bones, ligaments, tendons and nerves) | ⭘ | ⭘ | ⭘ |
| 4 | Rheumatoid Arthritis | ⭘ | ⭘ | ⭘ |
|  | **Metabolic** |  |  |  |
| 5 | Type 1 Diabetes | ⭘ | ⭘ | ⭘ |
| 6 | Type 2 Diabetes (T2DM) | ⭘ | ⭘ | ⭘ |
| 7 | Prediabetes (High blood sugar but not yet enough to be considered type 2 diabetes) | ⭘ | ⭘ | ⭘ |
| 8 | High cholesterol (Dyslipidemia) | ⭘ | ⭘ | ⭘ |
| 9 | Chronic Kidney Disease | ⭘ | ⭘ | ⭘ |
| 10 | Liver/Gallbladder/Pancreatic Disease – Non-alcoholic steatohepatitis (NASH)/Non-alcoholic fatty liver disease (NAFLD) | ⭘ | ⭘ | ⭘ |
| 11 | Kidney failure/Dialysis | ⭘ | ⭘ | ⭘ |
| 12 | Thyroid Disease | ⭘ | ⭘ | ⭘ |
|  | **Respiratory** |  |  |  |
| 13 | Asthma | ⭘ | ⭘ | ⭘ |
| 14 | Chronic obstructive pulmonary disease (COPD) (lung conditions causing breathing difficulties e.g. emphysema, chronic bronchitis) | ⭘ | ⭘ | ⭘ |
| 15 | Obstructive sleep apnea (OSA) | ⭘ | ⭘ | ⭘ |
|  | **Cardiovascular** |  |  |  |
| 16 | High blood pressure (Hypertension) | ⭘ | ⭘ | ⭘ |
| 17 | Hypertensive heart disease | ⭘ | ⭘ | ⭘ |
| 18 | Cerebrovascular Disease (Group of conditions, diseases and disorders that affect blood vessels and blood supply to the brain) | ⭘ | ⭘ | ⭘ |
| 19 | Coronary Heart Disease(Ischemic heart disease) | ⭘ | ⭘ | ⭘ |
| 20 | Heart failure | ⭘ | ⭘ | ⭘ |
| 21 | Cardiomyopathies (Diseases that affect the heart’s ability to pump blood around the body) | ⭘ | ⭘ | ⭘ |
| 22 | Deep vein thrombosis and pulmonary embolism (DVT and PE) | ⭘ | ⭘ | ⭘ |
| 23 | Cardiac arrest | ⭘ | ⭘ | ⭘ |
| 24 | Peripheral artery disease (Build up of fatty deposits in the arteries restricts blood supply to leg muscles) | ⭘ | ⭘ | ⭘ |
| 25 | Atrial fibrillation and flutter | ⭘ | ⭘ | ⭘ |
| 26 | Atherosclerosis (Arteries clogged with fatty substances) | ⭘ | ⭘ | ⭘ |
| 27 | I have a cardiovascular condition, but I am unsure which one | ⭘ | ⭘ | ⭘ |
|  | **Cancer** |  |  |  |
| 28 | Meningioma (A tumour that forms on the membranes that surround the brain and spinal cord) | ⭘ | ⭘ | ⭘ |
| 29 | Multiple myeloma | ⭘ | ⭘ | ⭘ |
| 30 | Esophageal cancer (cancer of the esophagus) | ⭘ | ⭘ | ⭘ |
| 31 | Cancers of the thyroid | ⭘ | ⭘ | ⭘ |
| 32 | Breast cancer | ⭘ | ⭘ | ⭘ |
| 33 | Gallbladder cancer | ⭘ | ⭘ | ⭘ |
| 34 | Stomach cancer | ⭘ | ⭘ | ⭘ |
| 35 | Liver cancer | ⭘ | ⭘ | ⭘ |
| 36 | Pancreatic cancer | ⭘ | ⭘ | ⭘ |
| 37 | Kidney cancer | ⭘ | ⭘ | ⭘ |
| 38 | Ovarian cancer | ⭘ | ⭘ | ⭘ |
| 39 | Uterine cancer | ⭘ | ⭘ | ⭘ |
| 40 | Colorectal cancer | ⭘ | ⭘ | ⭘ |
| 41 | Non-melanoma skin cancer | ⭘ | ⭘ | ⭘ |
| 42 | Other cancer | ⭘ | ⭘ | ⭘ |
|  | **Other** |  |  |  |
| 43 | PCOS (polycystic ovary syndrome) | ⭘ | ⭘ | ⭘ |
| 44 | Urinary incontinence | ⭘ | ⭘ | ⭘ |
| 45 | Psoriasis | ⭘ | ⭘ | ⭘ |
| 46 | Gastroesophageal reflux disease (GERD) (acid reflux) | ⭘ | ⭘ | ⭘ |
| 47 | Depression | ⭘ | ⭘ | ⭘ |
| 48 | Stomach ulcers (peptic ulcer) | ⭘ | ⭘ | ⭘ |
| 49 | Liver failure | ⭘ | ⭘ | ⭘ |
| 50 | Other condition(s) not listed above | ⭘ | ⭘ | ⭘ |

BQ7a. Please estimate the weight in Kg **[SHOW IN UK ONLY:** OR Stones and Lbs**]** that you were 12 months ago

**<Show Kg for all countries>**

**<Show all options for participants in UK; adjust question wording appropriately based on country>**

<NUMERIC & SINGLE CODE>

| ________________ | Kg <RANGE 1-500> | **OR** | ⭘Don't know |  |  |
| --- | --- | --- | --- | --- | --- |
| ________________ | Stones <RANGE: 1-99> | ________________ | Lbs <RANGE: 0-13> | **OR** | ⭘Don't know |

**<’Kg’, ‘Stones/Lbs’, and ‘don’t know’ are MUTUALLY EXCLUSIVE, show error message if values entered for both ‘*’Please provide a response in ‘Kg’, <OR ’Stones/Lbs’ <<UK only>>>, OR ‘don’t know*’>**

**<Show ‘Kg’ for all countries, only show Stones/Lbs for UK; adjust wording of question appropriately depending on country>**

**<’don’t know MUTUALLY EXCLUSIVE to numeric options>**

**<Prompt out of range error message: ‘’*Your response is not within the range we expected. If this response is correct, select ‘next’ to confirm, otherwise amend your response*’’>**

BQ7b. Please estimate how long you have been at or above the following weight: <<insert minimum weight needed for a BMI≥ 30 based on height entered at AQ5>>

If you have been at or above this weight for less than one year please state 0 years and then complete for the appropriate number of months.

<NUMERICAL; ASK ALL>

| ______________ | Years <RANGE: 0-100) | ______________ | Months <RANGE: 0-11) |
| --- | --- | --- | --- |

SECTION C: TREATMENT

**We would now like to ask you some questions about treatments you may have taken for your condition(s) within the last 12 months.**

CQ1a. You previously stated that you have been diagnosed with or treated for the following conditions.

**Have you taken a *prescription* medication for each of the following conditions within the last 12 months?**

PLEASE READ ALL OF THE GUIDANCE TEXT BEFORE ANSWERING THIS QUESTION

*Please only include medications that you receive a prescription for and that you can take/self-administer without the need to visit a health care practitioner/setting.*

*You may include treatments that you take with support from a caregiver.*

*Please do not include medications that you pay for that are not prescribed to you by a healthcare professional (e.g. a medication you purchase over the counter at a pharmacist without a prescription should not be included).*

|  |  | Yes | No | I don’t know if I received a prescription medication for this specific condition |
| --- | --- | --- | --- | --- |
|  | **Weight** |  |  |  |
| 1 | Weight Management/Reduction (Overweight or Obesity) | ⭘ | ⭘ | ⭘ |
|  | **Rheumatic diseases** |  |  |  |
| 2 | Osteoarthritis (OA) | ⭘ | ⭘ | ⭘ |
| 3 | Musculoskeletal pain (pain that affects the muscles, bones, ligaments, tendons and nerves) | ⭘ | ⭘ | ⭘ |
| 4 | Rheumatoid Arthritis | ⭘ | ⭘ | ⭘ |
|  | **Metabolic** |  |  |  |
| 5 | Type 1 Diabetes | ⭘ | ⭘ | ⭘ |
| 6 | Type 2 Diabetes (T2DM) | ⭘ | ⭘ | ⭘ |
| 7 | Prediabetes (High blood sugar but not yet enough to be considered type 2 diabetes) | ⭘ | ⭘ | ⭘ |
| 8 | High cholesterol (Dyslipidemia) | ⭘ | ⭘ | ⭘ |
| 9 | Chronic Kidney Disease | ⭘ | ⭘ | ⭘ |
| 10 | Liver/Gallbladder/Pancreatic Disease – Non-alcoholic steatohepatitis (NASH)/Non-alcoholic fatty liver disease (NAFLD) | ⭘ | ⭘ | ⭘ |
| 11 | Kidney failure/Dialysis | ⭘ | ⭘ | ⭘ |
| 12 | Thyroid Disease | ⭘ | ⭘ | ⭘ |
|  | **Respiratory** |  |  |  |
| 13 | Asthma | ⭘ | ⭘ | ⭘ |
| 14 | Chronic obstructive pulmonary disease (COPD) (lung conditions causing breathing difficulties e.g. emphysema, chronic bronchitis) | ⭘ | ⭘ | ⭘ |
| 15 | Obstructive sleep apnea (OSA) | ⭘ | ⭘ | ⭘ |
|  | **Cardiovascular** |  |  |  |
| 16 | High blood pressure (Hypertension) | ⭘ | ⭘ | ⭘ |
| 17 | Hypertensive heart disease | ⭘ | ⭘ | ⭘ |
| 18 | Cerebrovascular Disease (Group of conditions, diseases and disorders that affect blood vessels and blood supply to the brain) | ⭘ | ⭘ | ⭘ |
| 19 | Coronary Heart Disease(Ischemic heart disease) | ⭘ | ⭘ | ⭘ |
| 20 | Heart failure | ⭘ | ⭘ | ⭘ |
| 21 | Cardiomyopathies (Diseases that affect the heart’s ability to pump blood around the body) | ⭘ | ⭘ | ⭘ |
| 22 | Deep vein thrombosis and pulmonary embolism (DVT and PE) | ⭘ | ⭘ | ⭘ |
| 23 | Cardiac arrest | ⭘ | ⭘ | ⭘ |
| 24 | Peripheral artery disease (Build up of fatty deposits in the arteries restricts blood supply to leg muscles) | ⭘ | ⭘ | ⭘ |
| 25 | Atrial fibrillation and flutter | ⭘ | ⭘ | ⭘ |
| 26 | Atherosclerosis (Arteries clogged with fatty substances) | ⭘ | ⭘ | ⭘ |
| 27 | I have a cardiovascular condition, but I am unsure which one | ⭘ | ⭘ | ⭘ |
|  | **Cancer** |  |  |  |
| 28 | Meningioma (A tumour that forms on the membranes that surround the brain and spinal cord) | ⭘ | ⭘ | ⭘ |
| 29 | Multiple myeloma | ⭘ | ⭘ | ⭘ |
| 30 | Esophageal cancer (cancer of the esophagus) | ⭘ | ⭘ | ⭘ |
| 31 | Cancers of the thyroid | ⭘ | ⭘ | ⭘ |
| 32 | Breast cancer | ⭘ | ⭘ | ⭘ |
| 33 | Gallbladder cancer | ⭘ | ⭘ | ⭘ |
| 34 | Stomach cancer | ⭘ | ⭘ | ⭘ |
| 35 | Liver cancer | ⭘ | ⭘ | ⭘ |
| 36 | Pancreatic cancer | ⭘ | ⭘ | ⭘ |
| 37 | Kidney cancer | ⭘ | ⭘ | ⭘ |
| 38 | Ovarian cancer | ⭘ | ⭘ | ⭘ |
| 39 | Uterine cancer | ⭘ | ⭘ | ⭘ |
| 40 | Colorectal cancer | ⭘ | ⭘ | ⭘ |
| 41 | Non-melanoma skin cancer | ⭘ | ⭘ | ⭘ |
| 42 | Other cancer | ⭘ | ⭘ | ⭘ |
|  | **Other** |  |  |  |
| 43 | PCOS (polycystic ovary syndrome) | ⭘ | ⭘ | ⭘ |
| 44 | Urinary incontinence | ⭘ | ⭘ | ⭘ |
| 45 | Psoriasis | ⭘ | ⭘ | ⭘ |
| 46 | Gastroesophageal reflux disease (GERD) (acid reflux) | ⭘ | ⭘ | ⭘ |
| 47 | Depression | ⭘ | ⭘ | ⭘ |
| 48 | Stomach ulcers (peptic ulcer) | ⭘ | ⭘ | ⭘ |
| 49 | Liver failure | ⭘ | ⭘ | ⭘ |
| 50 | Other condition(s) not listed above | ⭘ | ⭘ | ⭘ |

<Pull through selected conditions from BQ6a. Always anchor code 50>

CQ1b. You mentioned that you have been prescribed medication for weight management/reduction (overweight or obesity) in the last 12 months.

**Who prescribed this medication for your weight management/reduction (overweight or obesity)?** <Show if ‘yes’ selected at code 1 CQ1a>

| 1 | 🞏 | General practitioner (GP)  SHOW AS POP UP **General practitioner definition**: A general practitioner (GP) is a doctor that treats all common medical conditions and includes doctors assistants, primary care nurses and internists (experts in internal organs) – these are the individuals you are likely to see before being referred to a specialist service. You may visit a general practitioner at a practice or clinic, an office in an outpatient hospital setting, a non-hospital building, a family practice or a standalone office |
| --- | --- | --- |
| 2 | 🞏 | Dietician  SHOW AS POP UP **Dietitian definition:** Dieticians are health care professionals that assess, diagnose and treat dietary and nutritional problems |
| 3 | 🞏 | Specialist physician  SHOW AS POP UP **Specialist physician definition**: Unlike a general practitioner a specialist doctor is a doctor that is trained and qualified to treat specific conditions – for example a bariatrician (weight management), cardiologist (heart conditions), dermatologist (skin conditions), gastroenterologist (digestive conditions), oncologist (cancer specialist) etc. You may visit a specialist physician at a practice or clinic, an office in a hospital setting, a non-hospital building or a standalone office. You are likely to have been referred to a specialist physician by another healthcare professional such as a general practitioner or following a visit to a hospital emergency department. |
| 4 | 🞏 | Nurse practitioner (HIDE IN FRANCE)  SHOW AS POP UP **Nurse practitioner definition:** Nurse practitioners are nurses who have additional education which increases their ability to treat, diagnose and prescribe medications to patients |
| 5 | 🞏 | (HIDE IN GERMANY)  Pharmacist – In person or online *(please only select if it was the pharmacist who prescribed the medication to you. Do not select if you only collected the medication from a pharmacist)* |
| 6 | 🞏 | Other |
| 7 | ⭘ | Don’t know <mutually exclusive with codes 1-6> |

CQ1c. In which healthcare setting was this medication prescribed to you? <Show if ‘yes’ selected at code 1 CQ1a>

| 1 | 🞏 | Primary care (general practitioners (GP, pharmacist etc.) |
| --- | --- | --- |
| 2 | 🞏 | Weight management/reduction service/clinic |
| 3 | 🞏 | Secondary care (e.g. hospital or specialist health care service/clinic) |
| 4 | 🞏 | Other |
| 5 | ⭘ | Don’t Know <mutually exclusive with codes 1-4> |

CQ2. You have stated that you have taken a prescription medication for each of the following conditions in the past 12 months.

**Please estimate how many different prescription medications you have taken for each condition in the last 12 months**.

Please only include prescribed medications that you can take/self-administer without the need to visit a health care practitioner/setting. You may include treatments that you take with support from a caregiver.

*For example, if you receive an intravenous cancer medication that you have to go to the hospital to receive, you do not need to include this when answering this question.*

<Show if ‘yes’ selected for at least one condition at CQ1a.>

PLEASE READ ALL OF THE GUIDANCE TEXT BEFORE ANSWERING THIS QUESTION

*If you have multiple conditions within one group of conditions shown below then please sum up all of the medications that you are taking for that group of conditions. For example, if you take two medications for high blood pressure and one medication for deep vein thrombosis then you would enter ‘3’ next to ‘Cardiovascular conditions’ below. If you then also took a medication for asthma, you would write ‘1’ next to ‘Respiratory conditions’ below.*

*If you are taking treatments that aren’t prescribed to you by your doctor, such as vitamins or occasional pain killers, please do not include these when answering this question*

|  |  | Number of prescription medications |
| --- | --- | --- |
| 1 | **Weight**  ((Pipe in condition selected at CQ1a code 1)) |  |
| 2 | **Rheumatic diseases**  ((Pipe in conditions selected at CQ1a codes 2 – 4)) |  |
| 3 | **Metabolic conditions**  ((Pipe in conditions selected at CQ1a codes 5 – 12)) |  |
| 4 | **Respiratory conditions**  ((Pipe in conditions selected at CQ1a codes 13 – 15)) |  |
| 5 | **Cardiovascular conditions**  ((Pipe in conditions selected at CQ1a codes 16 – 27)) |  |
| 6 | **Cancers**  ((Pipe in conditions selected at CQ1a codes 28 – 42)) |  |
| 7 | **Other conditions** ((Pipe in conditions selected at CQ1a codes 43 – 49)) |  |
| 8 | **Any other condition(s) not listed above** (If you are receiving prescription medications for more than one “other” condition please sum together the number of prescription medications you take for all other conditions not listed above) (Pipe in if selected code 50 at CQ1a ) |  |

<Only show conditions where ‘yes’ selected for at least one condition in that net at CQ1a>

<Range – 1-99>. >

<Show if ‘yes’ selected for at least one condition at CQ1a.>

CQ3. You have stated that you receive prescription medications for each of the conditions below.

**Thinking about each of these medications, please estimate how many times you received a prescription fill for each different condition in the last 12 months.**

Please only include prescribed medications that you can take/self-administer without the need to visit a health care practitioner/setting. You may include treatments that you take with support from a caregiver.

PLEASE READ ALL OF THE GUIDANCE TEXT BEFORE ANSWERING THIS QUESTION

*By ‘prescription fill’ we mean how many times you have received each medication in the last 12 months. This will include getting a medication for the first time (whether you picked it up from a pharmacy yourself or it was delivered to you either in hospital or at home) and any repeat prescriptions (refills) of the same medication.*

*For example, if person x was prescribed medication x by their doctor and picked it up from the pharmacist for the first time and then needed to receive a repeat for this medication two more times in the last 12 months, this would be a total of three prescription fills.*

*Please make sure you include all prescription fills for all of your medication. If you are taking more than one medication for the same condition, please include all prescription fills for all of those medications and sum them up before entering the number next to the condition below.*

|  |  | Number of times you have received a prescription fill | I don’t know how many times I have received a prescription fill for this specific condition |
| --- | --- | --- | --- |
| 1 | **Weight**  ((Pipe in condition selected at CQ1a code 1)) |  | ⭘ |
| 2 | **Rheumatic diseases**  ((Pipe in conditions selected at CQ1a codes 2 – 4)) |  | ⭘ |
| 3 | **Metabolic conditions**  ((Pipe in conditions selected at CQ1a codes 5 – 12)) |  | ⭘ |
| 4 | **Respiratory conditions**  ((Pipe in conditions selected at CQ1a codes 13 – 15)) |  | ⭘ |
| 5 | **Cardiovascular conditions**  ((Pipe in conditions selected at CQ1a codes 16 – 27)) |  | ⭘ |
| 6 | **Cancers**  ((Pipe in conditions selected at CQ1a codes 28 – 42)) |  | ⭘ |
| 7 | **Other conditions** ((Pipe in conditions selected at CQ1a codes 43 – 49)) |  | ⭘ |
| 8 | **Any other condition(s) not listed above** (If you received prescription fills for more than one “other” condition please sum together the number of prescription fills you have had for all other conditions not listed above) (Pipe in if selected code 50 at CQ1a) |  | ⭘ |

<Show conditions where ‘yes’ selected for at least one condition in that net at CQ1a>

<Range – 0-300>.

CQ4. You previously stated that you have been diagnosed with or treated for the following conditions.

**Have you received treatment that was administered by a health care practitioner in a health care setting for each of the following conditions within the last 12 months?**

PLEASE READ ALL OF THE GUIDANCE TEXT BEFORE ANSWERING THIS QUESTION

*Please only include treatments that required you to visit a health care setting/practitioner to receive the treatment. For example, an injection or an intravenous infusion (an IV or drip) that you visited a health care setting to receive. Other examples include radiotherapy and physiotherapy.*

|  |  | Yes | No | I don’t know if I received treatment that was administered by a health care practitioner in a health care setting for this specific condition |
| --- | --- | --- | --- | --- |
|  | **Weight** |  |  |  |
| 1 | Weight Management/Reduction (Overweight or Obesity) | ⭘ | ⭘ | ⭘ |
|  | **Rheumatic diseases** |  |  |  |
| 2 | Osteoarthritis (OA) | ⭘ | ⭘ | ⭘ |
| 3 | Musculoskeletal pain (pain that affects the muscles, bones, ligaments, tendons and nerves) | ⭘ | ⭘ | ⭘ |
| 4 | Rheumatoid Arthritis | ⭘ | ⭘ | ⭘ |
|  | **Metabolic** |  |  |  |
| 5 | Type 1 Diabetes | ⭘ | ⭘ | ⭘ |
| 6 | Type 2 Diabetes (T2DM) | ⭘ | ⭘ | ⭘ |
| 7 | Prediabetes (High blood sugar but not yet enough to be considered type 2 diabetes) | ⭘ | ⭘ | ⭘ |
| 8 | Dyslipidemia (High cholesterol) | ⭘ | ⭘ | ⭘ |
| 9 | Chronic Kidney Disease | ⭘ | ⭘ | ⭘ |
| 10 | Liver/Gallbladder/Pancreatic Disease – Non-alcoholic steatohepatitis (NASH)/Non-alcoholic fatty liver disease (NAFLD) | ⭘ | ⭘ | ⭘ |
| 11 | Kidney failure/Dialysis | ⭘ | ⭘ | ⭘ |
| 12 | Thyroid Disease | ⭘ | ⭘ | ⭘ |
|  | **Respiratory** |  |  |  |
| 13 | Asthma | ⭘ | ⭘ | ⭘ |
| 14 | Chronic obstructive pulmonary disease (COPD) (lung conditions causing breathing difficulties e.g. emphysema, chronic bronchitis) | ⭘ | ⭘ | ⭘ |
| 15 | Obstructive sleep apnea (OSA) | ⭘ | ⭘ | ⭘ |
|  | **Cardiovascular** |  |  |  |
| 16 | High blood pressure (Hypertension) | ⭘ | ⭘ | ⭘ |
| 17 | Hypertensive heart disease |  |  |  |
| 18 | Cerebrovascular Disease (Group of conditions, diseases and disorders that affect blood vessels and blood supply to the brain) | ⭘ | ⭘ | ⭘ |
| 19 | Coronary Heart Disease (Ischemic heart disease) | ⭘ | ⭘ | ⭘ |
| 20 | Heart failure | ⭘ | ⭘ | ⭘ |
| 21 | Cardiomyopathies (Diseases that affect the heart’s ability to pump blood around the body) | ⭘ | ⭘ | ⭘ |
| 22 | Deep vein thrombosis and pulmonary embolism (DVT and PE) | ⭘ | ⭘ | ⭘ |
| 23 | Cardiac arrest | ⭘ | ⭘ | ⭘ |
| 24 | Peripheral artery disease (Build up of fatty deposits in the arteries restricts blood supply to leg muscles) | ⭘ | ⭘ | ⭘ |
| 25 | Atrial fibrillation and flutter | ⭘ | ⭘ | ⭘ |
| 26 | Atherosclerosis (Arteries clogged with fatty substances) | ⭘ | ⭘ | ⭘ |
| 27 | I have a cardiovascular condition, but I am unsure which one | ⭘ | ⭘ | ⭘ |
|  | **Cancer** |  |  |  |
| 28 | Meningioma (A tumour that forms on the membranes that surround the brain and spinal cord) | ⭘ | ⭘ | ⭘ |
| 29 | Multiple myeloma | ⭘ | ⭘ | ⭘ |
| 30 | Esophageal cancer (cancer of the esophagus) | ⭘ | ⭘ | ⭘ |
| 31 | Cancers of the thyroid | ⭘ | ⭘ | ⭘ |
| 32 | Breast cancer | ⭘ | ⭘ | ⭘ |
| 33 | Gallbladder cancer | ⭘ | ⭘ | ⭘ |
| 34 | Stomach cancer | ⭘ | ⭘ | ⭘ |
| 35 | Liver cancer | ⭘ | ⭘ | ⭘ |
| 36 | Pancreatic cancer | ⭘ | ⭘ | ⭘ |
| 37 | Kidney cancer | ⭘ | ⭘ | ⭘ |
| 38 | Ovarian cancer | ⭘ | ⭘ | ⭘ |
| 39 | Uterine cancer | ⭘ | ⭘ | ⭘ |
| 40 | Colorectal cancer | ⭘ | ⭘ | ⭘ |
| 41 | Non-melanoma skin cancer | ⭘ | ⭘ | ⭘ |
| 42 | Other cancer | ⭘ | ⭘ | ⭘ |
|  | **Other** |  |  |  |
| 43 | PCOS (polycystic ovary syndrome) | ⭘ | ⭘ | ⭘ |
| 44 | Urinary incontinence | ⭘ | ⭘ | ⭘ |
| 45 | Psoriasis | ⭘ | ⭘ | ⭘ |
| 46 | Gastroesophageal reflux disease (GERD) (acid reflux) | ⭘ | ⭘ | ⭘ |
| 47 | Depression | ⭘ | ⭘ | ⭘ |
| 48 | Stomach ulcers (peptic ulcer) | ⭘ | ⭘ | ⭘ |
| 49 | Liver failure | ⭘ | ⭘ | ⭘ |
| 50 | Other condition(s) not listed above | ⭘ | ⭘ | ⭘ |

<Pull through selected conditions from BQ6a. Always anchor code 50>

CQ5. You have stated that you have received treatment that was administered by a health care practitioner at a health care setting for the following conditions.

**Please estimate the number of times that you received treatment that was administered by a health care practitioner in a health care setting for each of the following conditions within the last 12 months.**

PLEASE READ ALL OF THE GUIDANCE TEXT BEFORE ANSWERING THIS QUESTION

*Please only include treatments that required you to visit a health care setting/practitioner to receive the treatment. For example, an injection or an intravenous infusion (an IV or drip) that you visited a health care setting to receive. Other examples include radiotherapy and physiotherapy.*

|  |  | Number of times treatment was administered by a health care practitioner in a health care setting | I don’t know how many times treatment was administered by a health care practitioner in a health care setting |
| --- | --- | --- | --- |
| 1 | **Weight management/Reduction** (overweight or obesity) |  | ⭘ |
| 2 | **Rheumatic diseases**  ((Pipe in conditions selected at CQ4 codes 2 – 4)) |  | ⭘ |
| 3 | **Metabolic conditions**  ((Pipe in conditions selected at CQ4 codes 5 – 12)) |  | ⭘ |
| 4 | **Respiratory conditions**  ((Pipe in conditions selected at CQ4 codes 13 – 15)) |  | ⭘ |
| 5 | **Cardiovascular conditions**  ((Pipe in conditions selected at CQ4 codes 16 – 27)) |  | ⭘ |
| 6 | **Cancers**  ((Pipe in conditions selected at CQ4 codes 28 – 42)) |  | ⭘ |
| 7 | **Other conditions** ((Pipe in conditions selected at CQ4 codes 43 – 49)) |  | ⭘ |
| 8 | **Any other condition(s) not listed above** (If you received treatment administered by a health care practitioner in a healthcare setting for an “other” condition please sum together the number of times you have received this for all other conditions not listed above) (pipe in if code 50 selected at CQ4) |  | ⭘ |

<Show conditions where ‘yes’ selected for at least one condition in that net at CQ4>

<Range – 0-99>.

SECTION D: HEALTHCARE RESOURCE USE (LAST 12 MONTHS)

**Outpatient medical visits**

**We would like to ask you some questions about your medical history in the last 12 months.**

| DQ1ai. Have you visited or interacted with a **general practitioner** for any reason in the **last 12 months**?  *Please include* ***face to face*** *visits/interactions and* ***virtual*** *visits/interactions (e.g. telephone consultation, video call consultation etc.)* <SINGLE CODE>  **General practitioner definition**: A general practitioner (GP) is a doctor that treats all common medical conditions and includes doctors assistants, primary care nurses and internists (experts in internal organs) – these are the individuals you are likely to see before being referred to a specialist service. You may visit a general practitioner at a practice or clinic, an office in an outpatient hospital setting, a non-hospital building, a family practice or a standalone office | 1.⭘Yes, I have visited/interacted **face to face** **only**  2.⭘Yes, I have visited/interacted **virtually only**  3.⭘Yes, I have visited/interacted **both face to face and virtually**  4.⭘ No  5.⭘ I don’t know |
| --- | --- |
| DQ1aii. Please estimate how many **face to face general practitioner** **office visits** for any reason you have had in the **last 12 months**? < ASK if DQ1ai is code 1 or 3> <NUMERIC>  **General practitioner definition**: A general practitioner is a doctor that treats all common medical conditions and includes doctors assistants, primary care nurses and internists (experts in internal organs) – these are the individuals you are likely to see before being referred to a specialist service. You may visit a general practitioner at a practice or clinic, an office in an outpatient hospital setting, a non-hospital building, a family practice or a standalone office | ______________  <RANGE: 1-366> |
| DQ1aiii. Please estimate how many **virtual** (e.g. telephone consultation, video call consultation etc.) **general practitioner** **consultations** for any reason you have had in the **last 12 months**? < ASK if DQ1ai is code 2 or 3> <NUMERIC>  **General practitioner definition**: A general practitioner is a physician that treats all common medical conditions and includes physician’s assistants, primary care nurses and internists (expert in internal medicine) – these are the individuals you are likely to see before being referred to a specialist service. You may visit a general practitioner at a practice or clinic, an office in an outpatient hospital setting, a non-hospital building, a family practice or a standalone office | ______________  <RANGE: 1-366> |

| DQ2ai. Have you visited or interacted with a **specialist physician** for any reason in the **last 12 months**?  *Please include consultations that are* ***face to face*** *visits/interactions and* ***virtual*** *visits/interactions (e.g. telephone consultation, video call consultation etc. Please include specialist physician visits for routine consultations and assessments for any condition. Please do not include treatment related visits (captured in the previous section) or pre-operation (pre-op) assessment visits – you will be asked about this type of visit later.*  <SINGLE CODE>  **Specialist physician definition**: Unlike a general practitioner a specialist doctor is a doctor that is trained and qualified to treat specific conditions – for example a bariatrician (weight management), cardiologist (heart conditions), dermatologist (skin conditions), gastroenterologist (digestive conditions), oncologist (cancer specialist) etc. You may visit a specialist physician at a practice or clinic, an office in a hospital setting, a non-hospital building or a standalone office. You are likely to have been referred to a specialist physician by another healthcare professional such as a general practitioner or following a visit to a hospital emergency department.  **Definition of a pre-operation (pre-op) assessment meeting:**  Pre-operation assessment meetings are meetings that are conducted to prepare for a scheduled surgery. At this type of meeting you may be asked questions about your health, medical history, and home circumstances. If the assessment involves a visit to the hospital, some tests may be carried out. This is to check if you have any medical problems that might need to be treated before your operation, or if you'll need special care during or after the surgery. | 1.⭘Yes, I have visited/interacted **face to face** **only**  2.⭘Yes, I have visited/interacted **virtually only**  3.⭘Yes, I have visited/interacted **both face to face and virtually**  4.⭘ No  5.⭘ I don’t know |
| --- | --- |
| DQ2aii. Please estimate how many **face to face specialist physician** **office visits** for any reason you have had in the **last 12 months**? < ASK if DQ2ai is code 1 or 3> <NUMERIC>  **Specialist physician definition**: Unlike a general practitioner a specialist doctor is a doctor that is trained and qualified to treat specific conditions – for example a bariatrician (weight management), cardiologist (heart conditions), dermatologist (skin conditions), gastroenterologist (digestive conditions), oncologist (cancer specialist) etc. You may visit a specialist physician at a practice or clinic, an office in a hospital setting, a non-hospital building or a standalone office. You are likely to have been referred to a specialist physician by another healthcare professional such as a general practitioner or following a visit to a hospital emergency department. | ______________  <RANGE: 1-366> |
| DQ2aiii. Please estimate how many **virtual** (e.g. telephone consultation, video call consultation etc.) **specialist physician** **consultations** for any reason you have had in the **last 12 months**? < ASK if DQ2ai is code 2 or 3> <NUMERIC>  **Specialist physician definition**: Unlike a general practitioner a specialist doctor is a doctor that is trained and qualified to treat specific conditions – for example a bariatrician (weight management), cardiologist (heart conditions), dermatologist (skin conditions), gastroenterologist (digestive conditions), oncologist (cancer specialist) etc. You may visit a specialist physician at a practice or clinic, an office in a hospital setting, a non-hospital building or a standalone office. You are likely to have been referred to a specialist physician by another healthcare professional such as a general practitioner or following a visit to a hospital emergency department. | ______________  <RANGE: 1-366> |

**Inpatient hospitalisation and Emergency Department visits**

DQ3a. Have you had an **inpatient hospitalisation** for any reason in the **last 12 months**?

*Please count each individual admission as one admission even if the admission lasted for a number of days, do not count the number of days each admission lasted.* <ASK ALL>

**Inpatient hospitalization definition:** An admission to any department of a hospital that involved at least one overnight stay.

<SINGLE CODE>

| 1 | ⭘ | Yes |
| --- | --- | --- |
| 2 | ⭘ | No |
| 3 | ⭘ | I don’t know if I had an inpatient hospitalisation in the last 12 months |

DQ3b. Please estimate how many **inpatient hospitalisations** for any reason you have had in the **last 12 months**?

*Please count each individual admission as one admission even if the admission lasted for a number of days, do not count the number of days each admission lasted.*

**Inpatient hospitalization definition:** An admission to any department of a hospital that involved at least one overnight stay. <ASK IF CODE 1 for DQ3a>

< NUMERIC >

| ________________ | <RANGE: 1-366> |
| --- | --- |

DQ3c. You have reported that you had <<<insert response from DQ3b>>> **inpatient hospitalisations** in the **last 12 months**. <ASK IF CODE 1 for DQ3a

DQ3ci: How many of these admissions started with a visit to an **emergency department**? <ASK IF CODE 1 for DQ3a

DQ3cii: How many of these admissions that started with a visit to an **emergency department** required you to undergo a **surgical procedure/intervention**? <Show if DQ3ci >0>

**Inpatient hospitalization definition:** An admission to the hospital that involved at least one overnight stay.

< NUMERIC >

| DQ3ci | Number of inpatient admissions that included a visit to an emergency department | ________________ | <RANGE: 0 - max = response at DQ3b> |
| --- | --- | --- | --- |
| DQ3cii | Number of inpatient admissions that included a visit to an emergency department that required you to undergo a surgical procedure/intervention | ________________ | <RANGE: 0 – max = response at DQ3ci> |

DQ4a. Thinking about your <<<insert response from DQ3b>>> **inpatient hospitalisations**, please estimate how many nights **in total** you spent in hospital for any reason in the **last 12 months**? **Inpatient hospitalization definition:** An admission to the hospital that involved at least one overnight stay. <ASK IF CODE 1 FOR DQ3a >

< NUMERIC>

| ________________ | <RANGE: 1-366> |
| --- | --- |

<Ensure that value is greater than or equal to the value entered at DQ3b. If value is not greater than or equal to the value entered at DQ3b, show error message: ‘Value entered here is not greater than or equal to the number of inpatient hospitalizations you have recorded’>

DQ5a. Have you had an **outpatient emergency department visit** for any reason in the **last 12 months**? <ASK ALL>

**Outpatient emergency department visit definition:** a visit to an emergency department for the treatment of an accident or emergency that was completed without the need for you to be admitted to the hospital for an overnight stay. Please do not include any emergency department visits that resulted in an inpatient admission that you have just reported in the previous questions on inpatient admissions.

< SINGLE CODE>

| 1 | ⭘ | Yes |
| --- | --- | --- |
| 2 | ⭘ | No |
| 3 | ⭘ | I don’t know if I had an outpatient emergency room visit in the last 12 months |

DQ5b. Please estimate how many **outpatient emergency room department visits** for any reason you have had in the **last 12 months.** <ASK IF CODE 1 for DQ5a>

**Outpatient emergency department visit definition:** a visit to an emergency department for the treatment of an accident or emergency that was completed without the need for you to be admitted to the hospital for an overnight stay. Please do not include any emergency department visits that resulted in an inpatient admission that you have just reported for the previous questions on inpatient admissions.

< NUMERIC >

| ________________ | <RANGE: 1-366> |
| --- | --- |

| DQ6ai. Have you had a **scheduled surgical procedure/intervention** for any reason in the **last 12 months**? <SINGLE CODE>  **Definition of a scheduled surgical procedures/interventions**: A surgical procedure/intervention that you had a pre booked appointment for. | 1.⭘Yes  2.⭘ No  3.⭘ I don’t know |
| --- | --- |
| DQ6aii. Please estimate how many scheduled **surgical procedure/interventions** for any reason you have had in the **last 12 months**? <ASK if DQ6ai is code 1 > <NUMERIC>  **Definition of a scheduled surgical procedures/interventions**: A surgical procedure/intervention that you had a pre booked appointment for. | ______________  <RANGE: 1-366> |
| DQ6aiii. Please estimate how many of these scheduled **surgical procedure/interventions** were **inpatient surgeries** in the **last 12 months**? <ASK if DQ6ai is code 1 > <NUMERIC>  **Definition of an inpatient scheduled surgery**: A surgery that you had a pre booked appointment for that required at least one overnight stay in a health care setting. | ______________  <RANGE: 0-value at DQ6aii> |
| DQ6aiv. Please estimate how many of these scheduled **surgical procedure/interventions** were **outpatient surgeries** in the **last 12 months**? <ASK if DQ6ai is code 1 > <NUMERIC>  **Definition of an outpatient surgery:** A surgery that you had a pre booked appointment for that took place in an outpatient setting (you were able to return home the same day). To undergo this type of surgery you may have visited a hospital, a physician’s office, a non-hospital building, a practice or a clinic. | ______________  <RANGE: 0- value at DQ6aii >  <If value here summed with DQ6aiii does not total to DQ6aii show error message ‘*value entered here for outpatient surgeries, summed with value entered for inpatient surgeries (insert value from DQ6aii)i, does not sum to total number of scheduled surgeries you entered earlier (insert value from DQ6aii) please amend response*’ and allow participant to edit DQ6aiii and DQ6aiv> |
| DQ6av. Have you had any ***pre-operation (pre-op)*** assessment meetings for a scheduled surgical procedure/intervention in **the last 12 months**?  *Please include* ***face to face*** ***pre-operation (pre-op)*** *and* ***virtual*** *pre-op meetings (e.g. telephone consultation, video call consultation etc.) <SINGLE CODE>*  **Definition of a pre-operation (pre-op) assessment meeting :**  Pre-operation assessment meetings are meetings that are conducted to prepare for a scheduled surgery. At this type of meeting you may be asked questions about your health, medical history, and home circumstances. If the assessment involves a visit to the hospital, some tests may be carried out. This is to check if you have any medical problems that might need to be treated before your operation, or if you'll need special care during or after the surgery. | 1.⭘Yes, I have visited/interacted **face to face** **only**  2.⭘Yes, I have visited/interacted **virtually only**  3.⭘Yes, I have visited/interacted **both face to face and virtually**  4.⭘ No  5.⭘ I don’t know |
| DQ6avi. Please estimate how many **face to face *pre-operation (pre-op)*** **assessment meetings** you have had in the **last 12 months**? <ASK if DQ6av is code 1 or 3> <NUMERIC>  **Definition of a pre-operation (pre-op)** **assessment meeting :**  Pre-operation assessment meetings are meetings that are conducted to prepare for a scheduled surgery. At this type of meeting you may be asked questions about your health, medical history, and home circumstances. If the assessment involves a visit to the hospital, some tests may be carried out. This is to check if you have any medical problems that might need to be treated before your operation, or if you'll need special care during or after the surgery. | ______________  <RANGE: 1-366> |
| DQ6avii. Please estimate how many **virtual** (e.g. telephone consultation, video call consultation etc.) ***pre-operation (pre-op)*** **assessment meetings** you have had in the **last 12 months**? < ASK if DQ6av is code 2 or 3> <NUMERIC>  **Definition of a pre-operation (pre-op)** **assessment meeting:**  Pre-operation assessment meetings are meetings that are conducted to prepare for a scheduled surgery. At this type of meeting you may be asked questions about your health, medical history, and home circumstances. If the assessment involves a visit to the hospital, some tests may be carried out. This is to check if you have any medical problems that might need to be treated before your operation, or if you'll need special care during or after the surgery. | ______________  <RANGE: 1-366> |
| DQ6aviii. Have you had any follow up meetings for a scheduled surgical procedure/intervention in **the last 12 months**?  *Please include* ***face to face*** *follow up meetings and* ***virtual*** *follow up meetings (e.g. telephone consultation, video call consultation etc.) <SINGLE CODE>* | 1.⭘Yes, I have visited/interacted **face to face** **only**  2.⭘Yes, I have visited/interacted **virtually only**  3.⭘Yes, I have visited/interacted **both face to face and virtually**  4.⭘ No  5.⭘ I don’t know |
| DQ6avix. Please estimate how many **face to face follow up meetings** you have had in the **last 12 months**? <ASK if DQ6aviii is code 1 or 3> <NUMERIC> | ______________  <RANGE: 1-366> |
| DQ6avx. Please estimate how many **virtual** (e.g. telephone consultation, video call consultation etc.) **follow up meetings** you have had in the **last 12 months**? < ASK if DQ6aviii is code 2 or 3> <NUMERIC> | ______________  <RANGE: 1-366> |

DQ7a. Have you had a **laboratory pathology test** for any reason in the **last 12 months**? <ASK ALL>

**Definition of laboratory pathology tests**: A test conducted to diagnose or further understand a disease or condition, or conducted to monitor a condition over time. These tests typically include collecting a sample of blood, urine, or other body fluids (e.g. *complete blood count* (CBC – a blood test used to evaluate your overall health and detect a wide range of disorders), *glucose blood test* (a test that measures the glucose levels in your blood. Glucose is a type of sugar. It is your body's main source of energy), *bacterial culture of a tissue specimen* (a test to detect bacteria in for e.g. your skin etc), stool culture (testing your faeces), *urine bacteria culture*(test to detect bacteria in your urine), *hepatic function panel* (a blood test to check how well the liver is working), *lipid panel* (a blood test that measures lipids—fats and fatty substances used as a source of energy by your body for e.g. cholesterol)

<SINGLE CODE>

| 1 | ⭘ | Yes |
| --- | --- | --- |
| 2 | ⭘ | No |
| 3 | ⭘ | I don’t know if I had a laboratory and pathology test in the last 12 months |

DQ7b. Please estimate how many **laboratory pathology tests** for any reason you have had in the **last 12 months**? <ASK IF CODE 1 for DQ7a>

*If you have received tests for more than one condition please add together the number of tests for each condition and report the total number of tests. For example, patient x received 2 glucose blood tests to monitor their type 2 diabetes and 2 lipid panels to monitor their dyslipidemia so their total number of tests would be 4.*

**Definition of laboratory pathology tests**: A test conducted to diagnose or further understand a disease or condition, or conducted to monitor a condition over time. These tests typically include collecting a sample of blood, urine, or other body fluids (e.g. *complete blood count* (CBC – a blood test used to evaluate your overall health and detect a wide range of disorders), *glucose blood test* (a test that measures the glucose levels in your blood. Glucose is a type of sugar. It is your body's main source of energy), *bacterial culture of a tissue specimen* (a test to detect bacteria in for e.g. your skin etc), *stool culture* (testing your faeces), *urine bacteria culture* (test to detect bacteria in your urine), *hepatic function panel* (a blood test to check how well the liver is working), *lipid panel* (a blood test that measures lipids —fats and fatty substances used as a source of energy by your body for e.g. cholesterol)

< NUMERIC >

| ________________ | <RANGE: 1-999> |
| --- | --- |

DQ8a. Have you had a **radiology examination** for any reason in the **last 12 months**? <ASK ALL>

<SINGLE CODE>

**Definition of radiology examinations**: Radiology examinations may also be called diagnostic imaging. These examinations involve taking pictures or images of various parts of the body. Radiology exams involve radiation or other imaging procedures to find abnormalities and diagnose the cause of symptoms you may be experiencing; examples include a computed tomography scan (CT scan), ultrasound, X-rays, magnetic resonance imaging (MRI), bone density scans, mammograms, thyroid scans and fluoroscopy. You may also receive radiology examinations over time to monitor a condition.

| 1 | ⭘ | Yes |
| --- | --- | --- |
| 2 | ⭘ | No |
| 3 | ⭘ | I don’t know if I had a radiology examination in the last 12 months |

DQ8b. Please estimate how many **radiology examinations** for any reason you have had in the **last 12 months**? <ASK IF CODE 1 for DQ8a>

*If you have received examinations for more than one condition please add together the number of tests for each condition and report the total number of examinations. For example, patient x received 2 bone density scans and 2 x-rays to monitor their osteoarthritis and 1 MRI scan to check if they have a trapped nerve so their total number of examinations would be 5.*

**Definition of radiology examinations**: Radiology examinations may also be called diagnostic imaging. These examinations involve taking pictures or images of various parts of the body. Radiology exams involve radiation or other imaging procedures to find abnormalities and diagnose the cause of symptoms you may be experiencing; examples include a computed tomography scan (CT scan), ultrasound, X-rays, magnetic resonance imaging (MRI), bone density scans, mammograms, thyroid scans and fluoroscopy. You may also receive radiology examinations over time to monitor a condition.

< NUMERIC >

| ________________ | <RANGE: 1-999> |
| --- | --- |

DQ9a. Have you attempted to reduce your weight in the **last 12 months**? < ASK ALL>

<SINGLE CODE>

| 1 | ⭘ | Yes |
| --- | --- | --- |
| 2 | ⭘ | No |

DQ9b: What methods have you used to attempt to reduce your weight during the last 12 months?

|  |  | DQ9bi. **Please select all options that apply** <ASK IF CODE 1 for DQ9a> <MULTI CODE> | DQ9bii. **Was this funded publicly (i.e. funded/paid for by healthcare system) or privately (i.e. funded/paid for by health insurance or by yourself )?** <Show for selections at DQ9bi> <MULTI CODE; ‘Don’t know’ and ‘Not applicable’ SINGLE CODE & mutually exclusive with other options> <show soft prompt if select more than one option ‘*You have selected more than one option, please check your answer to ensure multiple selections are definitely correct*’> | DQ9biii. **Who was this weight reduction method recommended by?** <Show for selections at DQ9bi> <MULTI CODE; ‘don’t know’ SINGLE CODE & mutually exclusive with other options > |
| --- | --- | --- | --- | --- |
| 1 | 🞏 | I have taken a weight management/reduction (overweight or obesity) pharmaceutical treatment or medication | 🞏 Publicly (by the national health care system)  🞏 Privately (health insurance)  🞏 Privately (self-paid)  ⭘ Not applicable (i.e. no cost involved)  ⭘ Don't know  <*Must select a response(s) here if code 1 selected at CQ1a*> | 🞏 Self (independently decided to attempt this method)  🞏 General practitioner  🞏 Dietician  🞏 Specialist physician  🞏 Nurse  🞏 Pharmacist  🞏 Other  ⭘ Don't know |
| 2 | 🞏 | I have attempted a calorie controlled/restricted diet | 🞏 Publicly (by the national health care system)  🞏 Privately (health insurance)  🞏 Privately (self-paid)  ⭘ Not applicable (i.e. no cost involved)  ⭘ Don't know | 🞏 Self (independently decided to attempt this method)  🞏 General practitioner  🞏 Dietician  🞏 Specialist physician  🞏 Nurse  🞏 Pharmacist  🞏 Other  ⭘ Don't know |
| 3 | 🞏 | I have interacted with a weight loss service (for e.g. a commercial service such as Weight Watchers or a service provided by the national health service) | 🞏 Publicly (by the national health care system)  🞏 Privately (health insurance)  🞏 Privately (self-paid)  ⭘ Not applicable (i.e. no cost involved)  ⭘ Don't know | 🞏 Self (independently decided to attempt this method)  🞏 General practitioner  🞏 Dietician  🞏 Specialist physician  🞏 Nurse  🞏 Pharmacist  🞏 Other  ⭘ Don't know |
| 4 | 🞏 | I have joined a gym | 🞏 Publicly (by the national health care system)  🞏 Privately (health insurance)  🞏 Privately (self-paid)  ⭘ Not applicable (i.e. no cost involved)  ⭘ Don't know | 🞏 Self (independently decided to attempt this method)  🞏 General practitioner  🞏 Dietician  🞏 Specialist physician  🞏 Nurse  🞏 Pharmacist  🞏 Other  ⭘ Don't know |
| 5 | 🞏 | I have attempted to follow an exercise program/course (include online/virtual programs) | 🞏 Publicly (by the national health care system)  🞏 Privately (health insurance)  🞏 Privately (self-paid)  ⭘ Not applicable (i.e. no cost involved)  ⭘ Don't know | 🞏 Self (independently decided to attempt this method)  🞏 General practitioner  🞏 Dietician  🞏 Specialist physician  🞏 Nurse  🞏 Pharmacist  🞏 Other  ⭘ Don't know |
| 6 | 🞏 | I have undergone weight loss surgery | 🞏 Publicly (by the national health care system)  🞏 Privately (health insurance)  🞏 Privately (self-paid)  ⭘ Not applicable (i.e. no cost involved)  ⭘ Don't know | 🞏 Self (independently decided to attempt this method)  🞏 General practitioner  🞏 Dietician  🞏 Specialist physician  🞏 Nurse  🞏 Pharmacist  🞏 Other  ⭘ Don't know |
| 7 | 🞏 | I have used a digital health application for weight management | 🞏 Publicly (by the national health care system)  🞏 Privately (health insurance)  🞏 Privately (self-paid)  ⭘ Not applicable (i.e. no cost involved)  ⭘ Don't know | 🞏 Self (independently decided to attempt this method)  🞏 General practitioner  🞏 Dietician  🞏 Specialist physician  🞏 Nurse  🞏 Pharmacist  🞏 Other  ⭘ Don't know |
| 8 | 🞏 | I have undergone cognitive behavioral therapy (CBT) | 🞏 Publicly (by the national health care system)  🞏 Privately (health insurance)  🞏 Privately (self-paid)  ⭘ Not applicable (i.e. no cost involved)  ⭘ Don't know | 🞏 Self (independently decided to attempt this method)  🞏 General practitioner  🞏 Dietician  🞏 Specialist physician  🞏 Nurse  🞏 Pharmacist  🞏 Other  ⭘ Don't know |
| 9 | 🞏 | Alternative treatments for e.g. dietary supplements, herbal products etc. | 🞏 Publicly (by the national health care system)  🞏 Privately (health insurance)  🞏 Privately (self-paid)  ⭘ Not applicable (i.e. no cost involved)  ⭘ Don't know | 🞏 Self (independently decided to attempt this method)  🞏 General practitioner  🞏 Dietician  🞏 Specialist physician  🞏 Nurse  🞏 Pharmacist  🞏 Other  ⭘ Don't know |
| 10 | 🞏 | Other method not stated above | 🞏 Publicly (by the national health care system)  🞏 Privately (health insurance)  🞏 Privately (self-paid)  ⭘ Not applicable (i.e. no cost involved)  ⭘ Don't know | 🞏 Self (independently decided to attempt this method)  🞏 General practitioner  🞏 Dietician  🞏 Specialist physician  🞏 Nurse  🞏 Pharmacist  🞏 Other  ⭘ Don't know |

SECTION E: COVID-19

**The following questions refer to the COVID-19 (Coronavirus) outbreak.**

EQ1. Have you had symptoms of COVID-19 in the last 12 months? <ASK ALL>

< SINGLE CODE>

| 1 | ⭘ | Yes |
| --- | --- | --- |
| 2 | ⭘ | No |
| 3 | ⭘ | Don’t know |

EQ2. Have you had COVID-19 confirmed with a test in the last 12 months? <ASK ALL>

< SINGLE CODE>

| 1 | ⭘ | Yes |
| --- | --- | --- |
| 2 | ⭘ | No |
| 3 | ⭘ | Don’t know |

| EQ3a. Has COVID-19 affected your treatment for <pull through condition selected at BQ6a> <ASK ALL> <SINGLE CODE> in the last 12 months? | | | | |
| --- | --- | --- | --- | --- |
|  |  | <Condition 1 to show> | <condition 2 to show> | <condition 3 to show> |
| 1 | Yes | ⭘ | ⭘ | ⭘ |
| 2 | No | ⭘ | ⭘ | ⭘ |
| EQ3b. How has COVID-19 affected your treatment for <pull through condition selected at BQ6a> <ASK IF CODE 1 ‘YES’ FOR EQ3a> in the last 12 months? <must select 1 or more options in EQ3b for each condition where ‘yes’ selected in EQ3a | | | |  |
|  | <MULTI CODE> | <Condition 1 to show> | <condition 2 to show> | <condition 3 to show> |
| 1 | I have missed appointments because I had symptoms of COVID-19 | 🞏 | 🞏 | 🞏 |
| 2 | I have missed appointments due to fear of COVID-19 | 🞏 | 🞏 | 🞏 |
| 3 | My doctor has delayed or postponed appointments due to COVID-19 | 🞏 | 🞏 | 🞏 |
| 4 | My doctor has cancelled appointments due to COVID-19 | 🞏 | 🞏 | 🞏 |
| 5 | I have been unable to collect my drugs from the pharmacy | 🞏 | 🞏 | 🞏 |
| 6 | I have changed treatment | 🞏 | 🞏 | 🞏 |
| 7 | I have used less treatment to make it last longer | 🞏 | 🞏 | 🞏 |
| 8 | A scheduled surgery that I was due to undertake was delayed – please only include a surgery that was rescheduled and completed at a later date. | 🞏 | 🞏 | 🞏 |
| 9 | A scheduled surgery that I was due to take place was delayed or cancelled and has not yet been completed. | 🞏 | 🞏 | 🞏 |
| 10 | Face to face meetings with my doctor have been replaced by virtual (e.g. telephone consultation, video call consultation etc.) meetings | 🞏 | 🞏 | 🞏 |
| 11 | Other | 🞏 | 🞏 | 🞏 |
