# Supplementary material for: Obesity-related complications, healthcare resource use and weight loss strategies in six European countries: the RESOURCE survey
Source: Int J Obes (Lond). 2023 May 31;47(8):750–7. doi: 10.1038/s41366-023-01325-1 (PMC10359184; doi:10.1038/s41366-023-01325-1)
Supplement: Supplementary file 3 — Supplementary Table 1 [file 41366_2023_1325_MOESM3_ESM.docx]

## Supplementary Table S1. Summary and definitions of variables collected in the survey.

| **Variable group** | **Variable definition/concepts measured** | **Variable type** |
| --- | --- | --- |
| Screening  criteria | Age (years) | Categorical |
|  | Country of residence | Categorical |
|  | Current weight (kg/stones and lb [UK]) | Numeric |
|  | Current height (cm/feet and inches [UK]) | Numeric |
|  | Pregnancy status | Categorical |
|  | Healthcare use in last 12 months | Categorical |
| Respondent characteristics | Sex | Categorical |
|  | Age (years) | Numeric |
|  | Ethnic group | Categorical |
|  | Healthcare insurance | Categorical |
|  | Insurance coverage | Categorical |
|  | Smoking status | Categorical |
|  | Current diagnosed/treated conditions | Categorical |
|  | Time of diagnosis/treatment of conditions | Categorical |
|  | Weight 12 months ago (kg/stones and lb [UK]) | Numeric |
|  | Time at BMI ≥30 kg/m^2^ (years/months) | Numeric |
| Treatment | Treatment(s) prescribed (only those taken/self-administered without the need to visit a healthcare practitioner/setting) in the last 12 months (yes/no for each condition reported) | Categorical |
|  | Who prescribed medication for weight management/reduction (overweight or obesity) in the last 12 months | Categorical |
|  | Healthcare setting where medication for weight management/reduction (overweight or obesity) in the last 12 months was prescribed | Categorical |
|  | Number of prescriptions (only those taken/self-administered without the need to visit a healthcare practitioner/setting) for any condition in the last 12 months | Numeric |
|  | Number of times prescriptions filled (only those taken/self-administered without the need to visit a healthcare practitioner/setting) for any condition in the last 12 months | Numeric |
|  | Treatment(s) prescribed (only those administered by a healthcare practitioner in a healthcare setting) in the last 12 months (yes/no for each condition reported) | Categorical |
|  | Number of times treatment received (only those administered by a healthcare practitioner in a healthcare setting) for any condition in the last 12 months | Numeric |
| HCRU | Face-to-face/virtual GP visits in the last 12 months (yes/no) | Categorical |
|  | Number of face-to-face GP visits in the last 12 months | Numeric |
|  | Number of virtual GP visits in the last 12 months | Numeric |
|  | Total number of GP visits (face-to-face and virtual) in the last 12 months | Numeric |
|  | Face-to-face/virtual specialist visits in the last 12 months (yes/no) | Categorical |
|  | Number of face-to-face specialist visits in the last 12 months | Numeric |
|  | Number of virtual specialist visits in the last 12 months | Numeric |
|  | Total number of specialist visits (face-to-face and virtual) in the last 12 months | Numeric |
|  | Outpatient emergency room visits in the last 12 months (yes/no) | Categorical |
|  | Number of outpatient emergency room visits in the last 12 months | Numeric |
|  | Inpatient hospitalizations in the last 12 months (yes/no) | Categorical |
|  | Number of inpatient hospitalizations in the last 12 months | Numeric |
|  | Number of inpatient admissions that included a visit to an emergency department in the last 12 months | Numeric |
|  | Number of inpatient admissions that included a visit to an emergency department and required a surgical procedure/intervention in the last 12 months | Numeric |
|  | Total nights spent in hospital in the last 12 months | Numeric |
|  | Scheduled surgical procedures/interventions in the last 12 months (yes/no) | Categorical |
|  | Number of surgeries/interventions in the last 12 months | Numeric |
|  | Number of inpatient surgeries in the last 12 months | Numeric |
|  | Number of outpatient surgeries in the last 12 months | Numeric |
|  | Number of face-to-face pre-op meetings in the last 12 months | Numeric |
|  | Number of virtual pre-op meetings in the last 12 months | Numeric |
|  | Number of face-to-face follow-up meetings in the last 12 months | Numeric |
|  | Number of virtual follow-up meetings in the last 12 months | Numeric |
|  | Laboratory pathology tests in the last 12 months (yes/no) | Categorical |
|  | Number of laboratory pathology tests in the last 12 months | Numeric |
|  | Radiology examinations in the last 12 months (yes/no) | Categorical |
|  | Number of radiology examinations in the last 12 months | Numeric |
|  | Any other healthcare interaction in the last 12 months (yes/no) | Categorical |
|  | Type of other healthcare interaction in the last 12 months | Categorical |
|  | Weight loss attempted in the last 12 months | Categorical |
|  | Weight loss method attempted | Categorical |
|  | Funding (public or private) for weight loss method attempted | Categorical |
|  | Who was this weight reduction method recommended by? | Categorical |
| COVID-19 | Experienced symptoms of COVID-19 (yes/no) in the last 12 months | Categorical |
|  | Confirmation of COVID-19 diagnosis (yes/no) in the last 12 months | Categorical |
|  | Impact of COVID-19 on treatment for each condition in the last 12 months | Categorical |

BMI, body mass index; GP, general practitioner; HCRU, healthcare resource use.
