# Supplementary material for: Obesity-related complications, healthcare resource use and weight loss strategies in six European countries: the RESOURCE survey
Source: Int J Obes (Lond). 2023 May 31;47(8):750–7. doi: 10.1038/s41366-023-01325-1 (PMC10359184; doi:10.1038/s41366-023-01325-1)
Supplement: Supplementary file 4 — Supplementary Table 2 [file 41366_2023_1325_MOESM4_ESM.docx]

## Supplementary Table S2. Comorbidities recorded in the survey.

| **Weight** |
| --- |
| Weight management/reduction (overweight or obesity) |
| **Rheumatic diseases** |
| Osteoarthritis (OA) (a condition causing joint pain and stiffness) |
| Musculoskeletal pain (pain that affects the muscles, bones, ligaments, tendons and nerves) |
| Rheumatoid arthritis |
| **Metabolic conditions** |
| Type 1 diabetes |
| Type 2 diabetes (T2D) |
| Prediabetes (high blood sugar but not yet enough to be considered type 2 diabetes) |
| High cholesterol (dyslipidaemia) |
| Chronic kidney disease |
| Liver/gallbladder/pancreatic disease – non-alcoholic steatohepatitis (NASH)/non-alcoholic fatty liver disease (NAFLD) |
| Kidney failure/dialysis |
| Thyroid disease |
| **Respiratory conditions** |
| Asthma |
| Chronic obstructive pulmonary disease (COPD) (lung conditions causing breathing difficulties, e.g. emphysema, chronic bronchitis) |
| Obstructive sleep apnea (OSA) |
| **Cardiovascular conditions** |
| High blood pressure (hypertension) |
| Hypertensive heart disease |
| Cerebrovascular disease (group of conditions, diseases and disorders that affect blood vessels and blood supply to the brain) |
| Coronary heart disease (ischaemic heart disease) |
| Heart failure |
| Cardiomyopathies (diseases that affect the heart’s ability to pump blood around the body) |
| Deep vein thrombosis (DVT) and pulmonary embolism (PE) |
| Cardiac arrest |
| Peripheral artery disease (build-up of fatty deposits in the arteries restricts blood supply to leg muscles) |
| Atrial fibrillation and flutter |
| Atherosclerosis (arteries clogged with fatty substances) |
| I have a cardiovascular condition, but I am unsure which one |
| **Cancers** |
| Meningioma (a tumour that forms on the membranes that surround the brain and spinal cord) |
| Multiple myeloma |
| Oesophageal cancer (cancer of the oesophagus) |
| Cancers of the thyroid |
| Breast cancer |
| Gallbladder cancer |
| Stomach cancer |
| Liver cancer |
| Pancreatic cancer |
| Kidney cancer |
| Ovarian cancer |
| Uterine cancer |
| Colorectal cancer |
| Non-melanoma skin cancer |
| Other cancer |
| **Other conditions** |
| PCOS (polycystic ovary syndrome) |
| Urinary incontinence |
| Psoriasis |
| Gastro-oesophageal reflux disease (GERD) (acid reflux) |
| Depression |
| Stomach ulcers (peptic ulcers) |
| Liver failure |
| Other condition(s) not listed above |
| None of the above |
