# Supplementary material for: Obesity-related complications, healthcare resource use and weight loss strategies in six European countries: the RESOURCE survey
Source: Int J Obes (Lond). 2023 May 31;47(8):750–7. doi: 10.1038/s41366-023-01325-1 (PMC10359184; doi:10.1038/s41366-023-01325-1)
Supplement: Supplementary file 5 — Supplementary Table 3 [file 41366_2023_1325_MOESM5_ESM.docx]

## Supplementary Table S3. Demographic data, BMI and number of ORCs by country.

|  | **Total**  (*N* = 1850) | **France**  (*n* = 250) | **Germany**  (*n* = 250) | **Italy**  (*n* = 300) | **Spain** (*n* = 450) | **Sweden** (*n* = 100) | **UK** (*n* = 500) |
| --- | --- | --- | --- | --- | --- | --- | --- |
| **Age, years, *n* (%)** | | | | | | | |
| 18–34 | 205 (11.1) | 16 (6.4) | 19 (7.6) | 71 (23.7) | 67 (14.9) | 13 (13.0) | 19 (3.8) |
| 35–44 | 328 (17.7) | 38 (15.2) | 33 (13.2) | 79 (26.3) | 120 (26.7) | 18 (18.0) | 40 (8.0) |
| 45–54 | 417 (22.5) | 53 (21.2) | 53 (21.2) | 72 (24.0) | 130 (28.9) | 19 (19.0) | 90 (18.0) |
| 55–64 | 481 (26.0) | 83 (33.2) | 76 (30.4) | 47 (15.7) | 93 (20.7) | 28 (28.0) | 154 (30.8) |
| 65+ | 419 (22.6) | 60 (24.0) | 69 (27.6) | 31 (10.3) | 40 (8.9) | 22 (22.0) | 197 (39.4) |
| **Women, *n* (%)** | 963 (52.1) | 122 (48.8) | 109 (43.6) | 159 (53.0) | 259 (57.6) | 69 (69.0) | 245 (49.0) |
| **Ethnicity, *n* (%)** | | | | | | | |
| Caucasian | 1665 (90.0) | 221 (88.4) | 249 (99.6) | 242 (80.7) | 374 (83.1) | 99 (99.0) | 480 (96.0) |
| Hispanic | 42 (2.3) | 1 (0.4) | 0 (0) | 17 (5.7) | 24 (5.3) | 0 (0) | 0 (0) |
| Black/Afro-Caribbean | 15 (0.8) | 8 (3.2) | 0 (0) | 1 (0.3) | 1 (0.2) | 0 (0) | 5 (1.0) |
| Asian | 7 (0.4) | 1 (0.4) | 0 (0) | 0 (0) | 0 (0) | 1 (1.0) | 5 (1.0) |
| Other | 121 (6.5) | 19 (7.6) | 1 (0.4) | 40 (13.3) | 51 (11.3) | 0 (0) | 10 (2.0) |
| **Insurance type, *n* (%)** | | | | | | | |
| National health service | 1242 (67.1) | 46 (18.4) | 192 (76.8) | 225 (75.0) | 272 (60.4) | 78 (78.0) | 429 (85.8) |
| Private (self-paid) | 271 (14.6) | 113 (45.2) | 27 (10.8) | 56 (18.7) | 42 (9.3) | 9 (9.0) | 24 (4.8) |
| Private insurance, covered | 193 (10.4) | 4 (1.6) | 30 (12.0) | 9 (3.0) | 122 (27.1) | 0 (0) | 28 (5.6) |
| Other | 72 (3.9) | 63 (25.2) | 1 (0.4) | 1 (0.3) | 3 (0.7) | 0 (0) | 4 (0.8) |
| Don’t know | 72 (3.9) | 24 (9.6) | 0 (0) | 9 (3.0) | 11 (2.4) | 13 (13.0) | 15 (3.0) |
| **Obesity class,^a^ *n* (%)** | | | | | | | |
| Class I | 1042 (56.3) | 141 (56.4) | 115 (46.0) | 209 (69.7) | 260 (57.8) | 46 (46.0) | 271 (54.2) |
| Class II | 496 (26.8) | 69 (27.6) | 78 (31.2) | 63 (21.0) | 122 (27.1) | 29 (29.0) | 135 (27.0) |
| Class III | 312 (16.9) | 40 (16.0) | 57 (22.8) | 28 (9.3) | 68 (15.1) | 25 (25.0) | 94 (18.8) |
| **Number of ORCs, *n* (%)** | | | | | | | |
| 0 ORCs | 476 (25.7) | 60 (24.0) | 56 (22.4) | 98 (32.7) | 145 (32.2) | 24 (24.0) | 93 (18.6) |
| 1 ORC | 526 (28.4) | 86 (34.4) | 48 (19.2) | 78 (26.0) | 145 (32.2) | 31 (31.0) | 138 (27.6) |
| 2 ORCs | 362 (19.6) | 42 (16.8) | 55 (22.0) | 60 (20.0) | 82 (18.2) | 19 (19.0) | 104 (20.8) |
| ≥3 ORCs | 486 (26.3) | 62 (24.8) | 91 (36.4) | 64 (21.3) | 78 (17.3) | 26 (26.0) | 165 (33.0) |

^a^Class I: BMI 30 to <35 kg/m^2^; class II: BMI 35 to <40 kg/m^2^; class III: BMI 40 to <70 kg/m^2^.

BMI, body mass index; ORC, obesity-related complication.
