# Supplementary material for: Obesity-related complications, healthcare resource use and weight loss strategies in six European countries: the RESOURCE survey
Source: Int J Obes (Lond). 2023 May 31;47(8):750–7. doi: 10.1038/s41366-023-01325-1 (PMC10359184; doi:10.1038/s41366-023-01325-1)
Supplement: Supplementary file 6 — Supplementary Table 4 [file 41366_2023_1325_MOESM6_ESM.docx]

## Supplementary Table S4. Participants reporting ORCs in the past 12 months, by obesity class and number of ORCs.

|  | **Total**  (*N* = 1850) | **Obesity class I**  (*n* = 1042) | **Obesity class II**  (*n* = 496) | **Obesity class III**  (*n* = 312) | **1 ORC**  (*n* = 526) | **2 ORCs** (*n* = 362) | **≥3 ORCs** (*n* = 486) |
| --- | --- | --- | --- | --- | --- | --- | --- |
| Hypertension, *n* (%) | 727 (39.3) | 389 (37.3) | 199 (40.1) | 139 (44.6) | 159 (30.2) | 187 (51.7) | 381 (78.4) |
| Dyslipidaemia, *n* (%) | 422 (22.8) | 244 (23.4) | 106 (21.4) | 72 (23.1) | 39 (7.4) | 111 (30.7) | 272 (56.0) |
| Type 2 diabetes, *n* (%) | 323 (17.5) | 149 (14.3) | 93 (18.8) | 81 (26.0) | 47 (8.9) | 68 (18.8) | 208 (42.8) |
| Osteoarthritis, *n* (%) | 297 (16.1) | 150 (14.4) | 80 (16.1) | 67 (21.5) | 55 (10.5) | 69 (19.1) | 173 (35.6) |
| Musculoskeletal pain, *n* (%) | 252 (13.6) | 135 (13.0) | 67 (13.5) | 50 (16.0) | 48 (9.1) | 56 (15.5) | 148 (30.5) |
| Asthma, *n* (%) | 224 (12.1) | 127 (12.2) | 50 (10.1) | 47 (15.1) | 58 (11.0) | 58 (16.0) | 108 (22.2) |
| GERD, *n* (%) | 177 (9.6) | 97 (9.3) | 49 (9.9) | 31 (9.9) | 29 (5.5) | 42 (11.6) | 106 (21.8) |
| Obstructive sleep apnoea, *n* (%) | 160 (8.6) | 59 (5.7) | 54 (10.9) | 47 (15.1) | 16 (3.0) | 29 (8.0) | 115 (23.7) |
| Psoriasis, *n* (%) | 117 (6.3) | 60 (5.8) | 37 (7.5) | 20 (6.4) | 26 (4.9) | 25 (6.9) | 66 (13.6) |
| Urinary incontinence, *n* (%) | 97 (5.2) | 45 (4.3) | 28 (5.6) | 24 (7.7) | 13 (2.5) | 17 (4.7) | 67 (13.8) |
| Prediabetes, *n* (%) | 84 (4.5) | 49 (4.7) | 20 (4.0) | 15 (4.8) | 8 (1.5) | 22 (6.1) | 54 (11.1) |
| PCOS, *n* (%) | 62 (3.4) | 31 (3.0) | 18 (3.6) | 13 (4.2) | 18 (3.4) | 22 (6.1) | 22 (4.5) |
| ASCVD, *n* (%) | 71 (3.8) | 37 (3.6) | 21 (4.2) | 13 (4.2) | 3 (0.57) | 10 (2.8) | 58 (11.9) |
| CKD/kidney failure, *n* (%) | 40 (2.2) | 18 (1.7) | 10 (2.0) | 12 (3.8) | 4 (0.80) | 4 (1.1) | 32 (6.6) |
| Heart failure, *n* (%) | 32 (1.7) | 11 (1.1) | 13 (2.6) | 8 (2.6) | 3 (0.57) | 4 (1.1) | 25 (5.1) |

ASCVD, atherosclerotic cardiovascular disease; CKD, chronic kidney disease; GERD, gastro-oesophageal reflux disease; ORC, obesity-related complication; PCOS, polycystic ovary syndrome.
