# Supplementary material for: Obesity-related complications, healthcare resource use and weight loss strategies in six European countries: the RESOURCE survey
Source: Int J Obes (Lond). 2023 May 31;47(8):750–7. doi: 10.1038/s41366-023-01325-1 (PMC10359184; doi:10.1038/s41366-023-01325-1)
Supplement: Supplementary file 7 — Supplementary Table 5 [file 41366_2023_1325_MOESM7_ESM.docx]

## Supplementary Table S5. Participants reporting types of resource use in the past 12 months, by obesity class and number of ORCs.

|  | **Total**  (*N* = 1850) | **Obesity class I**  (*n* = 1042) | **Obesity class II**  (*n* = 496) | **Obesity class III**  (*n* = 312) | **0 ORCs**  (*n* = 476) | **1 ORC**  (*n* = 526) | **2 ORCs** (*n* = 362) | **≥3 ORCs** (*n* = 486) |
| --- | --- | --- | --- | --- | --- | --- | --- | --- |
| ≥1 inpatient admission, for any reason, *n* (%) | 334 (18.1) | 202 (19.4) | 78 (15.7) | 54 (17.3) | 64 (13.4) | 81 (15.4) | 59 (16.3) | 130 (26.7) |
| ≥1 inpatient admission including an ED visit, *n* (%) | 207 (11.2) | 133 (12.8) | 45 (9.1) | 29 (9.3) | 42 (8.8) | 46 (8.7) | 34 (9.4) | 95 (19.5) |
| ≥1 surgical procedure, for any reason, *n* (%) | 319 (17.2) | 183 (17.6) | 87 (17.5) | 49 (15.7) | 67 (14.1) | 83 (15.8) | 53 (14.6) | 116 (23.9) |
| ≥1 prescription treatment, *n* (%) | 1478 (79.9) | 810 (77.7) | 402 (81.0) | 266 (85.3) | 208 (43.7) | 455 (86.5) | 340 (93.9) | 475 (97.7) |
| ≥1 treatment administered by a healthcare professional in a healthcare setting, *n* (%) | 596 (32.2) | 311 (29.8) | 171 (34.5) | 114 (36.5) | 89 (18.7) | 169 (32.1) | 126 (34.8) | 212 (43.6) |

ED, emergency department; ORC, obesity-related complication.
