# Supplementary material for: Obesity-related complications, healthcare resource use and weight loss strategies in six European countries: the RESOURCE survey
Source: Int J Obes (Lond). 2023 May 31;47(8):750–7. doi: 10.1038/s41366-023-01325-1 (PMC10359184; doi:10.1038/s41366-023-01325-1)
Supplement: Supplementary file 8 — Supplementary Table 6 [file 41366_2023_1325_MOESM8_ESM.docx]

## Supplementary Table S6. Participants reporting weight loss strategies in the past 12 months, by obesity class and number of ORCs.

|  | **Total**  (*N* = 1454) | **Obesity class I**  (*n* = 807) | **Obesity class II**  (*n* = 397) | **Obesity class III**  (*n* = 250) | **0 ORCs**  (*n* = 370) | **1 ORC**  (*n* = 423) | **2 ORCs** (*n* = 283) | **≥3 ORCs** (*n* = 378) |
| --- | --- | --- | --- | --- | --- | --- | --- | --- |
| Calorie-controlled or restricted diet, *n* (%) | 1046 (71.9) | 580 (71.9) | 285 (71.8) | 181 (72.4) | 257 (69.5) | 294 (69.5) | 210 (74.2) | 285 (75.4) |
| Exercise programme or course, *n* (%) | 318 (21.9) | 184 (22.8) | 87 (21.9) | 47 (18.8) | 97 (26.2) | 93 (22.0) | 68 (24.0) | 60 (15.9) |
| Pharmaceutical treatment/medication, *n* (%) | 179 (12.3) | 92 (11.4) | 47 (11.8) | 40 (16.0) | 54 (14.6) | 48 (11.3) | 37 (13.1) | 40 (10.6) |
| Joined a gym, *n* (%) | 174 (12.0) | 97 (12.0) | 50 (12.6) | 27 (10.8) | 54 (14.6) | 52 (12.3) | 29 (10.2) | 39 (10.3) |
| Digital health application, *n* (%) | 141 (9.7) | 73 (9.0) | 43 (10.8) | 25 (10.0) | 44 (11.9) | 37 (8.7) | 26 (9.2) | 34 (9.0) |
| Alternative treatments, *n* (%) | 118 (8.1) | 59 (7.3) | 40 (10.1) | 19 (7.6) | 29 (7.8) | 29 (6.9) | 23 (8.1) | 37 (9.8) |
| Weight loss service, *n* (%) | 110 (7.6) | 46 (5.7) | 33 (8.3) | 31 (12.4) | 25 (6.8) | 36 (8.5) | 16 (5.7) | 33 (8.7) |
| Cognitive behavioural therapy, *n* (%) | 30 (2.1) | 12 (1.5) | 8 (2.0) | 10 (4.0) | 9 (2.4) | 4 (0.95) | 7 (2.5) | 10 (2.6) |
| Weight loss surgery, *n* (%) | 22 (1.5) | 10 (1.2) | 7 (1.8) | 5 (2.0) | 7 (1.9) | 5 (1.2) | 5 (1.8) | 5 (1.3) |
| Other, *n* (%) | 213 (14.6) | 120 (14.9) | 55 (13.9) | 38 (15.2) | 45 (12.2) | 64 (15.1) | 47 (16.6) | 57 (15.1) |

Digital health application refers to an application specifically for weight management. Alternative treatments include dietary supplements or herbal products. Weight loss service refers to both commercial services and programmes provided by the national health service.

ORC, obesity-related complication.
