# Supplementary material for: Obesity-related complications, healthcare resource use and weight loss strategies in six European countries: the RESOURCE survey
Source: Int J Obes (Lond). 2023 May 31;47(8):750–7. doi: 10.1038/s41366-023-01325-1 (PMC10359184; doi:10.1038/s41366-023-01325-1)
Supplement: Supplementary file 9 — Supplementary Table 7 [file 41366_2023_1325_MOESM9_ESM.docx]

## Supplementary Table S7. Weight loss by country and by obesity class.

|  | **France**  (*n* = 155) | **Germany**  (*n* = 187) | **Italy**  (*n* = 222) | **Spain** (*n* = 372) | **Sweden** (*n* = 73) | **UK** (*n* = 374) | **Obesity class I**  (*n* = 763) | **Obesity class II**  (*n* = 379) | **Obesity class III**  (*n* = 241) |
| --- | --- | --- | --- | --- | --- | --- | --- | --- | --- |
| **Threshold for clinically meaningful weight loss: ≥5%** | | | | | | | | | |
| Weight decreased, *n* (%) | 22 (14.2) | 46 (24.6) | 71 (32.0) | 112 (30.1) | 19 (26.0) | 97 (25.9) | 204 (26.7) | 102 (26.9) | 61 (25.3) |
| Weight remained the same, *n*(%) | 91 (58.7) | 107 (57.2) | 95 (42.8) | 161 (43.3) | 38 (52.1) | 208 (55.6) | 393 (51.5) | 187 (49.3) | 120 (49.8) |
| Weight increased, *n* (%) | 42 (27.1) | 34 (18.2) | 56 (25.2) | 99 (26.6) | 16 (21.9) | 69 (18.4) | 166 (21.8) | 90 (23.7) | 60 (24.9) |
| **Threshold for clinically meaningful weight loss: ≥10%** | | | | | | | | | |
| Weight decreased, *n* (%) | 6 (3.9) | 23 (12.3) | 37 (16.7) | 39 (10.5) | 4 (5.5) | 43 (11.5) | 82 (10.7) | 49 (12.9) | 21 (8.7) |
| Weight remained the same, *n*(%) | 125 (80.6) | 146 (78.1) | 157 (70.7) | 283 (76.1) | 62 (84.9) | 304 (81.3) | 600 (78.6) | 286 (75.5) | 191 (79.3) |
| Weight increased, *n* (%) | 24 (15.5) | 18 (9.6) | 28 (12.6) | 50 (13.4) | 7 (9.6) | 27 (7.2) | 81 (10.6) | 44 (11.6) | 29 (12.0) |
| **Threshold for clinically meaningful weight loss: ≥15%** | | | | | | | | | |
| Weight decreased, *n* (%) | 2 (1.3) | 11 (5.9) | 16 (7.2) | 18 (4.8) | 3 (4.1) | 18 (4.8) | 41 (5.4) | 21 (5.5) | 6 (2.5) |
| Weight remained the same, *n*(%) | 136 (87.7) | 165 (88.2) | 195 (87.8) | 330 (88.7) | 66 (90.4) | 347 (92.8) | 687 (90.0) | 337 (88.9) | 215 (89.2) |
| Weight increased, *n* (%) | 17 (11) | 11 (5.9) | 11 (5.0) | 24 (6.5) | 4 (5.5) | 9 (2.4) | 35 (4.6) | 21 (5.5) | 20 (8.3) |
