# Supplementary material for: Obesity-related complications, healthcare resource use and weight loss strategies in six European countries: the RESOURCE survey
Source: Int J Obes (Lond). 2023 May 31;47(8):750–7. doi: 10.1038/s41366-023-01325-1 (PMC10359184; doi:10.1038/s41366-023-01325-1)
Supplement: Supplementary file 10 — Supplementary Information [file 41366_2023_1325_MOESM10_ESM.docx]

**Titles and descriptions for supplemental files**

**Supplementary methods**

Title: Survey design, development and quality control

Description: Additional methodological detail on survey development

Format: Word

**Supplementary file – RESOURCE survey**

Title: RESearch survey assessing individuals with Obesity to Understand their healthcare Resource use and Characteristics within the EU5 and Sweden (RESOURCE)

Description: Document containing full survey questions and details

Format: Word

Supplementary Table 1.

Title: Summary and definitions of variables collected in the survey.

Description: Full list of variables collected in the survey

Format: Word

Supplementary Table 2.

Title: Comorbidities recorded in the RESOURCE survey.

Description: Full list of comorbidities recorded in the RESOURCE survey

Format: Word

**Supplementary Table 3.**

Title: Demographic data, BMI and number of ORCs by country.

Description: Demographic and clinical characteristics of survey participants, stratified by country.

Format: Word

**Supplementary Table 4.**

Title: Participants reporting ORCs in the past 12 months, by obesity class and number of ORCs.

Description: Full numbers and percentages of patients reporting specific obesity-related complications (ORCs), stratified by obesity class and number of ORCs

Format: Word

**Supplementary Table 5.**

Title: Participants reporting types of resource use in the past 12 months, by obesity class and number of ORCs.

Description: Full numbers and percentages of patients reporting different types of healthcare resource use, stratified by obesity class and number of ORCs

Format: Word

**Supplementary Table 6.**

Title: Participants reporting weight loss strategies in the past 12 months, by obesity class and number of ORCs.

Description: Full numbers and percentages of patients reporting different weight loss strategies, stratified by obesity class and number of ORCs

Format: Word

**Supplementary Table 7.**

Title: Weight loss by country and by obesity class.

Description: Full numbers and percentages of patients reporting different degrees of weight loss, stratified by country and obesity class

Format: Word
